# Peripheral immune cells in metastatic breast cancer patients display a systemic immunosuppressed signature consistent with chronic inflammation

**DOI:** 10.1038/s41523-024-00638-2

**Published:** 2024-04-23

**Authors:** Sudhir Kumar Chauhan, Claire Dunn, Nikolai Kragøe Andresen, Andreas Hagen Røssevold, Gjertrud Skorstad, Adam Sike, Bjørnar Gilje, Sunil Xavier Raj, Kanutte Huse, Bjørn Naume, Jon Amund Kyte

**Affiliations:** 1 Department of Cancer Immunology, Institute for Cancer Research, Oslo University Hospital, Oslo, Norway; 2 Department of Clinical Cancer Research, Oslo University Hospital, Oslo, Norway; 3 Institute of Clinical Medicine, University of Oslo, Oslo, Norway; 4 Department of Hematology and Oncology, Stavanger University Hospital, Stavanger, Norway; 5 Department of Oncology, St Olav University Hospital, Trondheim, Norway; 6 Department of Oncology, Oslo University Hospital, Oslo, Norway; 7 Faculty of Health Sciences, Oslo Metropolitan University, Oslo, Norway

**Keywords:** Breast cancer, Tumour immunology, Biomarkers

## Abstract

Immunotherapies blocking the PD-1/PD-L1 checkpoint show some efficacy in metastatic breast cancer (mBC) but are often hindered by immunosuppressive mechanisms. Understanding these mechanisms is crucial for personalized treatments, with peripheral blood monitoring representing a practical alternative to repeated biopsies. In the present study, we performed a comprehensive mass cytometry analysis of peripheral blood immune cells in 104 patients with HER2 negative mBC and 20 healthy donors (HD). We found that mBC patients had significantly elevated monocyte levels and reduced levels of CD4^+^ T cells and plasmacytoid dendritic cells, when compared to HD. Furthermore, mBC patients had more effector T cells and regulatory T cells, increased expression of immune checkpoints and other activation/exhaustion markers, and a shift to a Th2/Th17 phenotype. Furthermore, T-cell phenotypes identified by mass cytometry correlated with functionality as assessed by IFN-γ production. Additional analysis indicated that previous chemotherapy and CDK4/6 inhibition impacted the numbers and phenotype of immune cells. From 63 of the patients, fresh tumor samples were analyzed by flow cytometry. Paired PBMC-tumor analysis showed moderate correlations between peripheral CD4^+^ T and NK cells with their counterparts in tumors. Further, a CD4^+^ T cell cluster in PBMCs, that co-expressed multiple checkpoint receptors, was negatively associated with CD4^+^ T cell tumor infiltration. In conclusion, the identified systemic immune signatures indicate an immune-suppressed environment in mBC patients who had progressed/relapsed on standard treatments, and is consistent with ongoing chronic inflammation. These activated immuno-suppressive mechanisms may be investigated as therapeutic targets, and for use as biomarkers of response or treatment resistance.

## Introduction

Breast cancer (BC) is a leading cause of cancer-related deaths and in 2020 surpassed lung carcinoma as the most commonly diagnosed cancer worldwide, with an estimated 2.3 million new cases^1^. BC in the early stage is curable in ~70–80% of patients^2^, but despite new therapeutic interventions, metastatic BC remains incurable with a median overall survival of less than 3 years^3^. BC is a heterogeneous disease, routinely classified by the expression of hormone receptors (HR) and Human epidermal growth factor receptor 2 (HER2). In the present study, we investigate peripheral blood immune cells in patients with metastatic HR^+^HER2^−^ BC (HR^+^ BC) or HR^−^HER2^−^ BC (triple-negative BC). While the HR^+^ HER2^−^ subgroup is the most common type of BC, triple-negative BC (TNBC) represents only 10–15% of patients^4^. However, the prognosis for metastatic TNBC is poor, with limited therapeutic options^5^.

Metastatic BC is a systemic disease that influences the circulating immune cells and can lead to systemic immune irregularities. In addition, therapeutic agents may modulate the immune system. Thus, peripheral immune profiles could serve as useful and non-invasive clinical biomarkers for predicting treatment response and monitoring disease progression. However, the majority of studies in BC patients have focused on total peripheral blood counts, neutrophil/lymphocyte/monocytes ratios (NLR and LMR)^6–8^, or on selected major immune subsets without extensive phenotypic determination^9–14^. Many of these studies did not include healthy individuals and there is limited information on how immune phenotypes differ between healthy individuals and metastatic BC patients. It is well known that TNBC and HR^+^ BC differ widely in their underlying biology and sensitivity to treatments, however, differences in their peripheral immune subsets have not been characterized. A detailed phenotypic characterization of different immune cell populations in the peripheral blood of metastatic BC patients will help in identifying their utility in clinical monitoring and interpreting their relevance as biomarkers for future targeted clinical trials.

The selection of adjuvant BC therapy is generally standardized. However, a physician is often presented with several alternatives for treating metastatic BC, with limited biomarkers to enable treatment selection. Tumor infiltrating lymphocytes (TIL) are associated with better prognosis and therapeutic response, especially in TNBC^15–17^, and the use of immunotherapy against TNBC is based on assessment of Programmed Cell Death Protein Ligand 1 (PD-L1) in tumor biopsies^18^. However, these and other facets of the tumor microenvironment are known to change over time^19,20^, especially during the development of treatment resistance. As repeated biopsies of metastatic lesions are often not feasible, less invasive approaches for monitoring tumor-immune co-evolution would be invaluable. Further, the immune milieu in BC differs from melanoma and other cancer forms where immune checkpoint inhibitors have been most effective. An improved understanding of the expression of targets like immune checkpoints in metastatic BC may offer leads for designing effective immunotherapies.

We addressed the above-mentioned need for improved knowledge by performing comprehensive immune profiling of peripheral blood mononuclear cells (PBMCs) from metastatic BC patients and healthy donors by mass cytometry. Here, we present the results of simultaneous phenotypic analysis of lymphoid and myeloid immune subsets from metastatic BC in comparison to healthy controls and relate our findings to BC subtypes and previous received treatments. We further characterized the T cell phenotypes in metastatic BC patients and investigated their association with T cell functionality. Moreover, we characterized the immune cells in tumor biopsies and compared this to the PBMC signatures. Our findings will provide a valuable resource of peripheral immune cell status of metastatic BC patients for future preclinical and clinical studies.

## Results

### Comparison of immune cell composition in BC patients and healthy donors

**Fig. 1:**
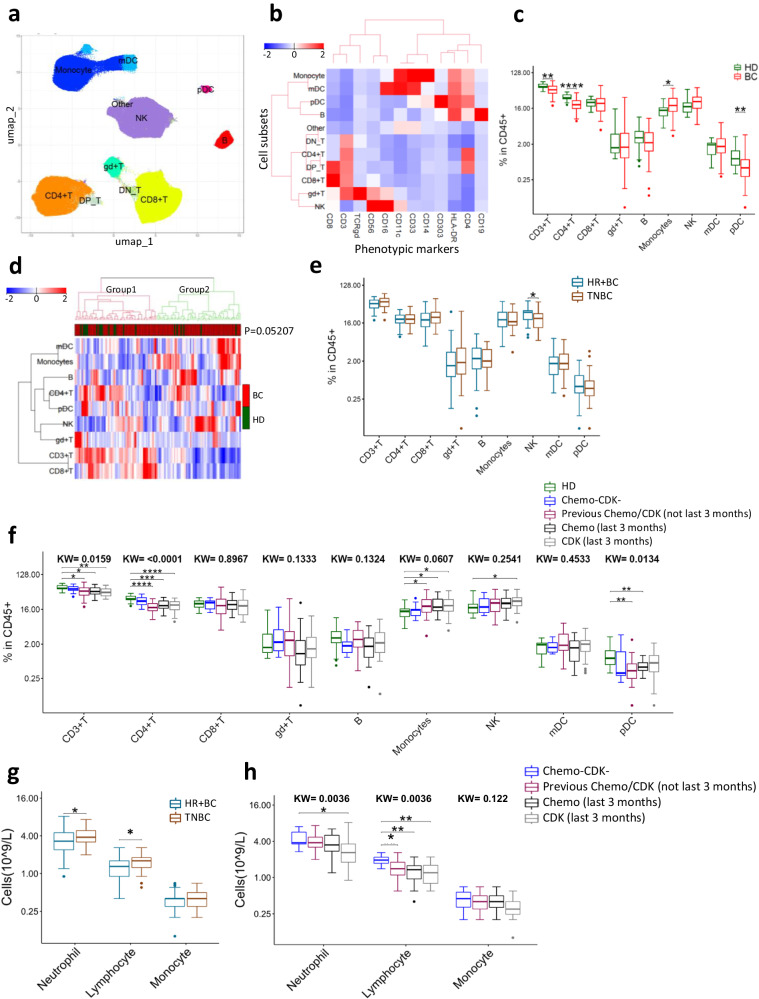
Mass cytometry analysis of major immune subsets in peripheral blood of breast cancer (BC) patients and healthy donors (HD). **a** UMAP analysis of PBMCs using 12 phenotypic markers (CD3, CD4, CD8, TCRgd, CD19, CD56, CD16, CD33, CD14, CD11c, HLA-DR, and CD303). Different immune subsets were identified by phenograph clustering. **b** Heat map depicting mean expression of phenotypic markers on different immune subsets. **c** Abundance of lymphocyte and myeloid immune cell subsets in BC patients (*n* = 104) and HD (*n* = 20). **d** Heat map representing hierarchical clustering of all samples based on abundance of different immune subsets. Groups were compared with Fisher’s exact test. **e** Abundance of lymphocyte and myeloid cell subsets in patients with HR^+^ BC (*n* = 57) or TNBC (*n* = 47). **f** Effect of previous therapy on immune cells**:** Received no chemotherapy/CDK inhibitors (Chemo^−^CDK^−^; *n* = 10), received chemotherapy/CDK inhibitors > 3 months before sample collection [Previous Chemo/CDK (not last 3 months); *n* = 40], received chemotherapy within 3 months before sample collection [Chemo (last 3 months); n = 26], received CDK inhibitors within 3 months before sample collection [CDK (last 3 months); *n* = 28]. **g** Absolute numbers of immune cell subsets per liter blood of HR^+^ BC (*n* = 57) and TNBC patients (*n* = 47). **h** Absolute numbers of immune cell subsets per liter blood of patients grouped based on previous treatments. Two groups were compared by Wilcoxon Mann–Whitney Rank Sum Test. More than two groups were compared by Kruskal–Wallis test, followed by pair-wise comparisons using Wilcoxon Mann–Whitney Rank Sum Test using HD (Chemo^−^CDK^−^ for Fig. 1h) as a reference group. FDR-adjusted *p*-value (p.adj) <0.05 was considered statistically significant. *p.adj < 0.05, **p.adj < 0.01, ***p.adj < 0.001, ****p.adj < 0.0001. Box plots show median (center line), 25th and 75th percentiles (box), data within 1.5 IQR (whiskers), and outliers (dots). *Abbreviations:* T, T cells, B, B cells, NK Natural Killer cells, mDC myeloid dendritic cells, pDC plasmacytoid dendritic cells, DN double negative, DP double positive.

The patients’ characteristics and clinical details are provided in Table 1. PBMCs from 104 patients with metastatic HER2^−^ BC and 20 HD were analyzed by single cell mass cytometry, and the .fcs files from each sample subjected to dimensionality reduction using the Uniform Manifold Approximation and Projection (UMAP) algorithm. This was followed by the identification of immune cell clusters by PhenoGraph utilizing a set of 12 core phenotypic markers (CD3, CD4, CD8, TCR-γδ, CD19, CD56, CD16, CD14, CD33, HLA-DR, CD11c, and CD303). This approach led to unique cell clusters [(CD4^+^ T, CD8^+^ T), gamma delta T (γδ^+^ T), B cells, natural killer (NK) cells, Monocytes, myeloid dendritic cells (mDCs), and plasmacytoid dendritic cells (pDCs)] as shown in Fig. 1a, b.

**Table 1:** Characteristics of breast cancer patients included in the study

	All (*n* = 104)	TNBC (*n* = 47)	HR^+^ BC (*n* = 57)
Age (years)	54.0 (47.5-62.0)	55.0 (43.0-62.0)	53.0 (48.0-62.0)
Sex			
* Male*	1 (1.0%)	0 (0.0%)	1 (1.8%)
* Female*	103 (99.0%)	47 (100.0%)	56 (98.2%)
Breast cancer subtype			
* Triple negative BC (TNBC)*	47 (45.2%)		
* HR*^+^*/HER2*^−^ *BC (HR*^+^ *BC)*	57 (54.8%)		
Denovo metastatic disease	25 (24%)	11 (23.4%)	14 (24.6%)
Disease stage at sampling			
* Stage IV*	104 (100.0%)	47 (100.0%)	57 (100.0%)
Previous (neo)adjuvant chemotherapy	74 (71.2%)	35 (74.5%)	39 (68.4%)
Previous Lines of metastatic chemotherapy			
*None*	56 (53.8%)	29 (61.7%)	27 (47.4%)
* 1 line*	46 (44.2%)	17 (36.2%)	29 (50.9%)
* 2 lines*	2 (1.9%)	1 (2.1%)	1 (1.8%)
Chemotherapy naive	17 (16.3%)	9 (19.1%)	8 (14.0%)
Previous CDK4/6 inhibitor	55 (52.9%)	3 (6.4%)	52 (91.2%)
Previous adjuvant radiotherapy	74 (71.2%)	29 (61.7%)	45 (78.9%)
Previous palliative radiotherapy	30 (28.8%)	9 (19.1%)	21 (36.8%)
Initial diagnosis to sampling (months)	63.4 (26.6-120.4)	31.2 (12.9-86.7)	83.2 (44.0-145.0)
Advanced diagnosis to sampling (months)	16.9 (2.1-30.0)	1.9 (0.8-7.2)	24.8 (17.5-37.5)
Bone metastases	72 (69.2%)	21 (44.7%)	51 (89.5%)
Liver metastases	61 (58.7%)	17 (36.2%)	44 (77.2%)
Lung metastases	34 (32.7%)	20 (42.6%)	14 (24.6%)
More than 3 sites of metastases	22 (21.2%)	7 (14.9%)	15 (26.3%)

Data are presented as median (IQR) for continuous measures, and *n* (%) for categorical measures.

To validate the UMAP-PhenoGraph guided approach of classifying immune cells, we also performed manual gating (gating scheme, Supplementary Fig. 1c), and found that the UMAP-PhenoGraph guided immune cell classification correlated well with the manual gating (Supplementary Fig. 1e). When we compared these major immune subsets between BC patients and HD, we found that BC patients had significantly higher percentage of monocytes and lower percentages of CD4^+^ T and pDCs compared to HD (Fig. 1c). NK cell levels were also higher in BC patients but not statistically significant (*p* = 0.054). No significant difference was observed for CD8^+^ T, γδ^+^ T, B cells and mDCs (Fig. 1c). Hierarchical clustering (Fig. 1d) revealed that HD clustered mostly in group 1, characterized by high numbers of T cells and lower levels of monocytes. However, this association was not statistically significant (Fisher’s exact test, *p* = 0.052).

To identify the differences in immune profile based on the clinically relevant BC subtypes, we classified the patients into HR^+^ BC and TNBC. Both groups had comparable frequencies of immune cells in PBMCs except for NK cells which were significantly higher in HR^+^ BC (Fig. 1e). Moreover, we calculated the absolute number of neutrophils, lymphocytes, and monocytes in whole blood, based on differential counts from the same time point as the PBMCs used for mass cytometry. The TNBC patients had higher neutrophil and lymphocyte counts compared to HR^+^ BC (Fig. 1g).

### The impact of therapy on PBMC composition

We further investigated how the immune cell composition was influenced by previous treatment with chemotherapeutic agents or Cyclin D Kinase 4/6-inhibitors (CDKi), both of which give clinically important hematological toxicity, and whether the observed differences between HD and BC patients could be attributed to previous therapy. To this aim, we compared the immune profile of HD with BC patients who had never received chemotherapy or CDKi (*n* = 10), patients who received chemotherapy/CDKi more than 3 months before sample collection (*n* = 40) and patients who received chemotherapy/CDKi within the last 3 months (*n* = 54). The distribution and characteristics of HR^+^ BC and TNBC patients in these treatment groups are shown in Supplementary Table 1. There were no significant differences in immune cell proportions between HD and untreated patients, while patients with current or previous chemotherapy/CDKi therapy had lower CD4^+^ T cell and pDC levels, as well as a higher percentage of monocytes (Fig. 1f). These data thus suggested that the observed differences between HD and patients could be related to the treatment, rather than to the disease. Interestingly, there were no significant differences between currently treated and previously treated patients, indicating that treatment-induced changes persisted over time. Patients receiving treatments had lower lymphocyte counts compared to untreated patients irrespective of when last treatment was given (Fig. 1h). The median counts for both neutrophils and monocytes were lower in patients recently treated with CDK inhibitors, though only reaching statistical significance for neutrophils (Fig. 1h).

### Increased effector memory CD4^+^ T cells in BC patients

**Fig. 2:**
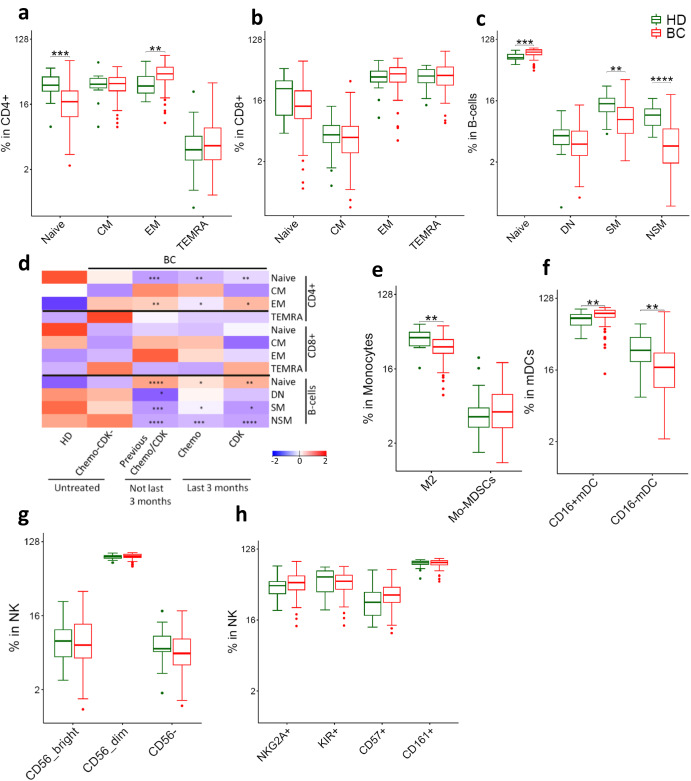
Differentiation subsets of immune cells in BC patients and HD. **a**–**d** Adaptive immune cells (**a**) CD4^+^ T (**b**) CD8^+^ T (**c**) B-cells (**d**) Effect of previous chemotherapy and CDK inhibitors on phenotype of adaptive immune cells in BC patients. Heatmap represents relative median of each cell subset across HD and different patient groups. **e**–**h** Innate immune cells (**e**) Monocytes (**f**) mDCs (**g**-**h**) NK cells. Two groups were compared by Wilcoxon Mann-Whitney Rank Sum Test. More than two groups were compared by kruskal wallis test, followed by pair-wise comparisons using Wilcoxon Mann–Whitney Rank Sum Test with HD as a reference group. *p.adj* < 0.05 was considered statistically significant. *p.adj < 0.05, **p.adj < 0.01, ***p.adj < 0.001, ****p.adj < 0.0001. BC: *n* = 104. HD: *n* = 20. Box plots show median (center line), 25th and 75th percentiles (box), data within 1.5 IQR (whiskers), and outliers (dots). *Abbreviations:* CM central memory, EM effector memory, TEMRA effector memory re-expressing CD45RA, SM switched memory, NSM non-switched memory, DN double negative, Mo-MDSCs Monocytic myeloid derived suppressor cells.

Next, we analyzed the relative abundance of the major T-cell and B-cell differentiation subsets (gating strategy Supplementary Fig. 2). T cells are classically sub-grouped into naive, central memory (CM), effector memory (EM) and effector memory re-expressing CD45RA (TEMRA). We found that BC patients had significantly higher EM CD4^+^ T cells and reduced naive CD4^+^ T cells than healthy donors (Fig. 2a, b). This was more pronounced in chemotherapy/CDKi treated patients (Fig. 2d and Supplementary Fig. 3a, b). The comparison of HD to untreated patients did not reach to statistical significance but followed the same trend as for treated patients (Fig. 2d, and Supplementary Fig. 3a, b). The composition of the T cell subtypes in HR^+^ BC and TNBC patients were largely similar (Supplementary Fig. 4a, b).

BC patients also had a skewed B-cell phenotype compared to HD, with a significantly higher level of naive B cells, and fewer memory B cells (Fig. 2c). This observation appeared to be influenced by therapy, as patients with previous chemotherapy or CDKi treatment had more naive but less non-switched memory (NSM) and switched memory (SM) B cells, whereas untreated patients did not (Fig. 2d, Supplementary Fig. 3c).

### Lower levels of M2 monocytes and CD16^−^ mDCs in BC patients

We further analyzed key subsets of innate immune cells (gating strategy Supplementary Fig. 2). Myeloid-derived suppressor cells (MDSCs) and M2 (CD163^+^ ) monocytes are reported to be important immunosuppressive cells in cancer^21^. In our data set, the MDSC-levels were similar in BC patients and HD. Unexpectedly, we found that the M2 monocytes levels were lower in patients (Fig. 2e). The M2 monocytes and MDSC levels were generally comparable across treatment groups (Supplementary Fig. 3d) and were also similar between TNBC and HR^+^ BC patients (Supplementary Fig. 4d). Since MDSC exerts their pro-tumor effect by suppressing T cells, we further analyzed the MDSC to T-cells ratio in HD and BC patients. MDSC to T-cells ratio was significantly higher in BC patients compared to HD (Supplementary Fig. 5a). Furthermore, BC patients receiving treatments had higher MDSC to T-cells ratio compared to HD (Supplementary Fig. 5b).

mDCs can be divided into two subsets based on CD16 expression. CD16^−^ DCs represent the classical mDCs with superior antigen presentation capacity, whereas CD16^+^ mDCs have a transcriptional program resembling CD16^+^ monocytes. BC patients had reduced CD16^−^ mDCs compared to HD (Fig. 2f). This observation could not be attributed to therapy, as the two mDC subsets were comparable among the treatment groups (Supplementary Fig. 3e). The mDC composition was similar in TNBC and HR^+^ BC patients (Supplementary Fig. 4e).

NK cells have potent anti-tumor activity. There were no differences in the major NK cell subsets between HD and BC patients (Fig. 2g). However, BC Patients had slightly higher numbers of the terminally differentiated, highly cytotoxic CD57^+^ NK cells (p.adj >0.05; Fig. 2h) that was independent of chemotherapy/CDKi treatment (Supplementary Fig. 3g). The NK phenotype was largely similar between HR^+^ BC and TNBC patients (Supplementary Fig. 4f, g).

### T cells from BC patients exhibit activated/exhausted phenotype

**Fig. 3:**
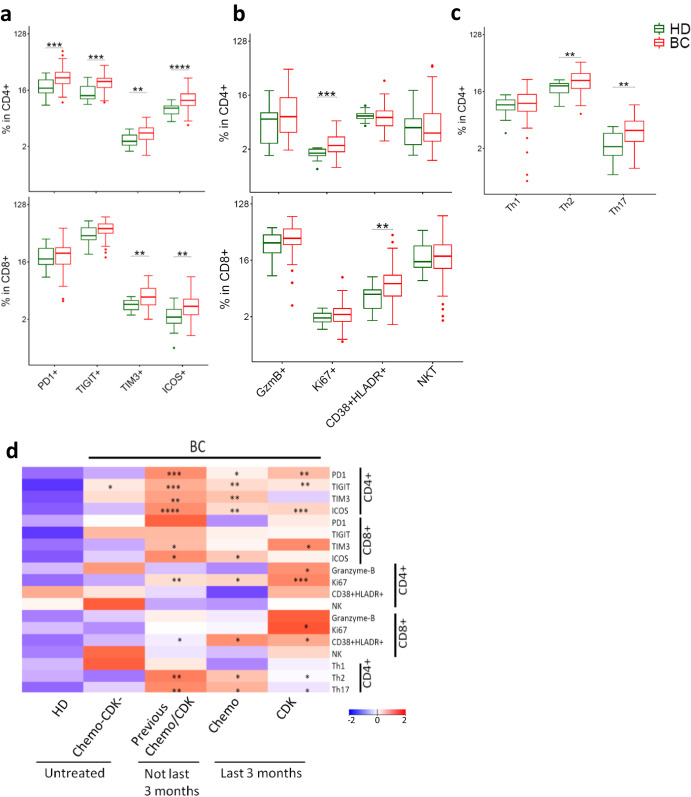
Phenotypic characterization of T cells in BC patients and HD. Expression of immune checkpoint receptors (**a**) and functional/phenotypic markers (**b**) in CD4^+^ and CD8^+^ T cells. **c** Abundance of Th1/Th2/Th17 subsets in CD4^+^ T cells. **d** Effect of previous chemotherapy and CDK inhibitors on phenotype of T cells in BC patients. Heatmap represents relative median of each cell subset across HD and different patient groups. Two groups were compared by Wilcoxon Mann–Whitney Rank Sum Test. More than two groups were compared by Kruskal–Wallis test, followed by pair-wise comparisons using Wilcoxon Mann-Whitney Rank Sum Test using HD as a reference group. *p.adj < 0.05, **p.adj < 0.01, ***p.adj < 0.001, ****p.adj < 0.0001. BC: *n* = 104. HD: *n* = 20. Box plots show median (center line), 25th and 75th percentiles (box), data within 1.5 IQR (whiskers), and outliers (dots). *Abbreviations:* NKT natural killer T cells.

The findings described above suggested a shift in the T cell population in BC patients compared to HD, with a decrease in total T cells accompanied by increased EM and decreased naive T cell subsets. Therefore, we further characterized the T cell phenotype, focusing on markers associated with anti-tumor immunity, in the form of immune checkpoint receptors (PD1, TIGIT, TIM3, ICOS) and other markers of T cell functionality (Granzyme-B, Ki67, Th-subsets) (gating strategy Supplementary Fig. 6). The results showed that BC patients expressed significantly higher levels of TIM3 and ICOS on both CD4^+^ and CD8^+^ T cells, whereas PD1 and TIGIT were significantly increased only on CD4^+^ T cells (Fig. 3a). BC patients had significantly more Ki67^+^ CD4^+^ T cells, suggesting ongoing T cell proliferation, and had more CD38^+^HLADR^+^ cells in the CD8 compartment (Fig. 3b). Most of these differences were more pronounced in patients treated with chemotherapy/CDKi, compared to untreated patients (Fig. 3d, Supplementary Fig. 7) and were independent of BC subtypes (Supplementary Fig. 8).

Among CD4^+^ T cells, a Th1-phenotype is considered desirable for anti-tumor immunity. The Th1/Th2/Th17-subsets are generally identified by cytokine profile, but the surface proteins CXCR3, CCR4, and CCR6 are also commonly used to identify phenotypes suggestive of these subsets. In our dataset, these phenotypic markers suggested that BC patients had more Th2 and Th17 cells than HD, whereas the frequency of Th1 cells was similar between HD and BC patients (Fig. 3c).

### Unsupervised analyses identified differentially expressed T cell clusters in patients

**Fig. 4:**
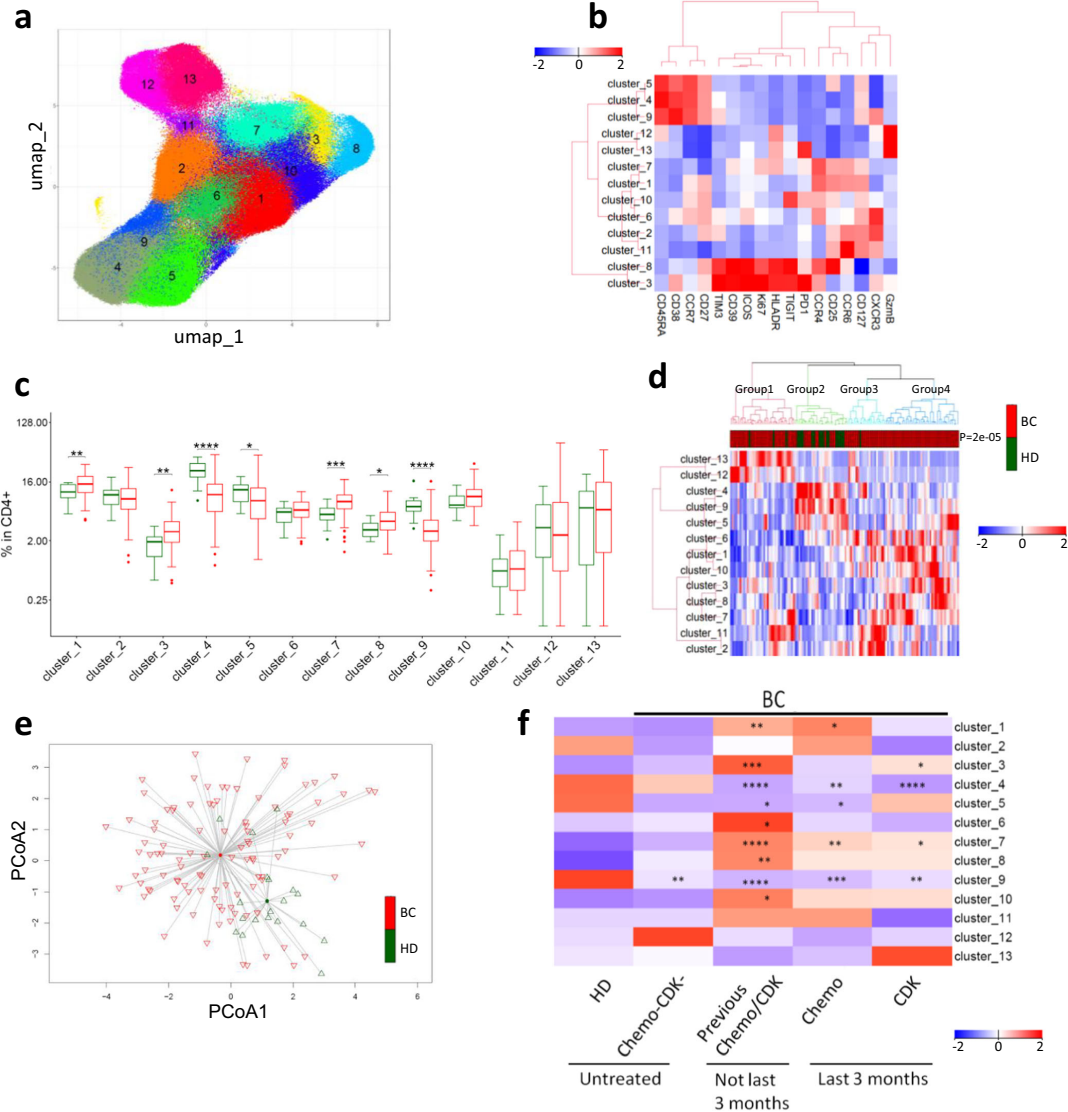
Unsupervised analysis identified CD4^+^ T cell clusters with different abundance in patients and HD. **a** CD4^+^ T cells from patients and HD were subjected to UMAP followed by automated clustering using phenograph. 13 unique clusters were identified. **b** Heat map showing expression of phenotypic/functional markers in CD4^+^ T cell clusters. **c** Comparative abundance of CD4^+^ T cell clusters between HD and BC patients. Groups were compared by Wilcoxon Mann-Whitney Rank Sum Test. **d** Heat map representing hierarchical clustering of all samples based on CD4^+^ T cell clusters. Proportions of each cluster were scaled and centered. Groups were compared with Fisher’s exact test. **e** Principal coordinate analysis (PCoA) of BC patients and HD using all 13 CD4^+^ T cell clusters. **f** Effect of previous therapy on identified CD4^+^ T cell clusters. Heatmap represents relative median of each cell subset. *p.adj < 0.05, **p.adj < 0.01, ***p.adj < 0.001, ****p.adj < 0.0001. Box plots show median (center line), 25th and 75th percentiles (box), data within 1.5 IQR (whiskers), and outliers (dots). BC: *n* = 104. HD: *n* = 20.

We next performed an unsupervised analysis of CD4^+^ T cells in BC patients using UMAP dimensionality reduction followed by automated clustering with PhenoGraph. This approach led to identification of 13 unique CD4^+^ cell clusters (Fig. 4a, b). Eight of these CD4^+^ clusters were significantly dysregulated in BC patients. Clusters 1, 3, 7, and 8 were upregulated in BC, while clusters 4, 5, 9 were downregulated (Fig. 4c). A closer inspection of the dysregulated CD4 clusters revealed that the downregulated clusters (4, 5 and 9) were all naive CD4 T cells. These were mostly negative for immune checkpoints, and activation and proliferation markers (Fig. 4b). The upregulated clusters 3, and 8 included Tregs (Cluster 8) and T cells positive for multiple immune checkpoint receptors (Cluster 3), whereas the upregulated clusters 1 and 7 were negative for immune checkpoints but expressed CCR4 and CCR6 (Fig. 4b). Hierarchical clustering and PART-guided classification of BC patients and HD led to 4 subgroups, with most of the HD confined to group 2 (*p* = 0.0001) (Fig. 4d). Further principal coordinate analysis of PhenoGraph identified CD4^+^ T cell clusters revealed that HD clustered separately from BC patients (Fig. 4e). In line with findings described above, most of these differences were more pronounced in patients treated with chemotherapy/CDKi compared (Fig. 4f, Supplementary Fig. 7d). The findings in TNBC and HR^+^ BC patients were largely similar (Supplementary Fig. 8d).

### Increased and activated Tregs in BC patients

**Fig. 5:**
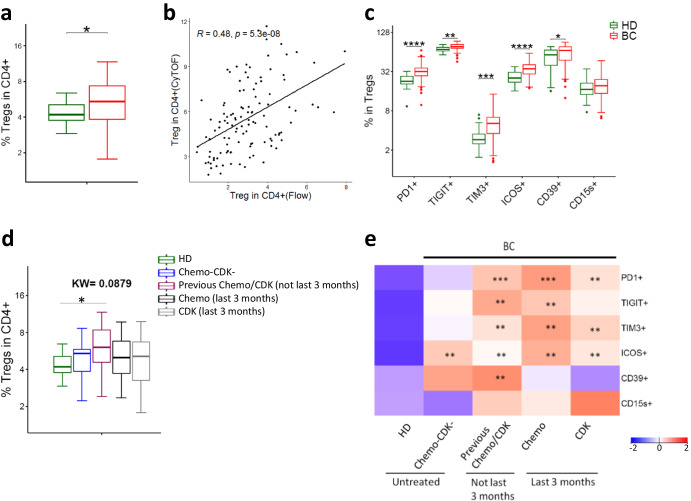
Phenotypic characterization of regulatory T cells (Tregs) in BC patients. **a** Tregs (CD25^+^CD127^−^) in CD4 compartment identified by mass cytometry. **b** Correlation between Tregs identified by CyTOF (CD25^+^CD127^−^) and Flow cytometry (CD25^+^Foxp3^+^) (BC = 99, HD = 16). Spearman rank correlation. **c** Expression of immune checkpoints and functional markers on Tregs. **d** Effect of previous chemotherapy and CDK inhibitors on abundance of Tregs. **e** Effect of previous therapy on expression of immune checkpoints and functional markers on Tregs. Heatmap represents relative median of each cell subset across HDs and different patient groups. Two groups were compared by Wilcoxon Mann-Whitney Rank Sum Test. More than two groups were compared by Kruskal–Wallis test, followed by pair-wise comparisons using Wilcoxon Mann-Whitney Rank Sum Test with HD as a reference group. *p.adj < 0.05, **p.adj < 0.01, ***p.adj < 0.001, ****p.adj < 0.0001. Box plots show median (center line), 25th and 75th percentiles (box), data within 1.5 IQR (whiskers), and outliers (dots).

The identification of increased Tregs by automated clustering (CD25^+^CD127^−^CD4^+^) (Cluster 8, Fig. 4b, c) prompted us to look at these cells in more detail. Manual gating (CD25^+^CD127^−^CD4^+^ ; gating strategy Supplementary Fig. 6a) produced results consistent with the UMAP/PhenoGraph-guided approach (Fig. 5a). Since we did not have Foxp3 in our mass cytometry panel, we also analyzed Tregs (CD4^+^CD25^+^Foxp3^+^) by flow cytometry (gating strategy Supplementary Fig. 9) to further confirm our findings. Treg levels determined by mass cytometry correlated well with Flow cytometry estimates (Fig. 5b). Like CD4^+^ T cells, Tregs from BC patients expressed high levels of immune checkpoint receptors (PD1, TIGIT, TIM3, and ICOS) (Fig. 5c). In addition, they also expressed more CD39 compared to HD (Fig. 5c). Treg numbers and phenotype were largely comparable between treatment groups and independent of BC subtype (Fig. 5d, e and Supplementary Fig. 10).

### IFNγ production correlates with mass cytometry identified T cell subsets

**Fig. 6:**
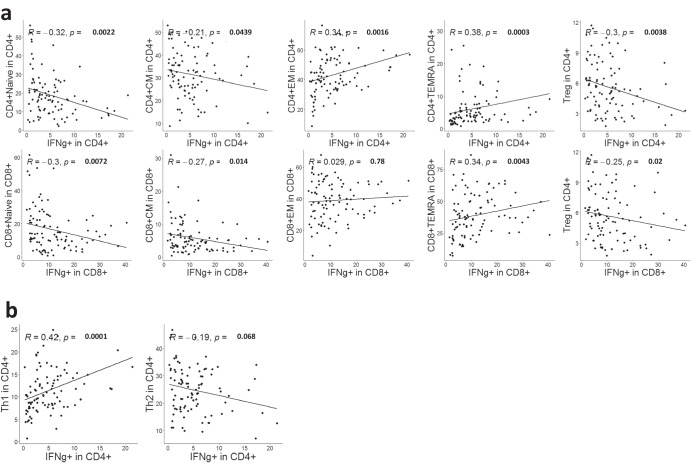
IFNγ production correlates with T cell subsets identified by manual gating. PBMCs from BC patients (*n* = 95) were stimulated by CD3/CD28 beads. IFNγ production was determined by flow cytometry. **a** Correlation of IFNγ production by CD4 (upper) and CD8 (lower) T cells with differentiation subsets of T cells (Naive, CM, EM, and TEMRA) and Tregs. **b** Correlation of IFNγ production by CD4 (upper) and CD8 (lower) T cells with Th subsets identified by CyTOF. Spearman rank correlation. FDR-adjusted *p*-values are shown.

To investigate the functional capacity of T cells in BC patients and evaluate their correlation with immune subsets identified by mass cytometry, we stimulated PBMCs from BC patients with human T cell activator CD3/CD28 DynaBeads, and measured IFNγ production by flow cytometry. IFNγ production correlated positively with the proportion of EM CD4^+^ T cells and TEMRA T cells, and correlated negatively with CM T cells, naive T cells and Tregs (Fig. 6a). These observations suggest that the mass cytometry-based phenotyping correlated well to T cell functionality, as it is expected that EM cells produce more cytokines upon stimulation than naïve cells, and that Tregs suppress T cell function and do not produce IFNγ. Furthermore, we investigated how IFNγ production correlated with the Th-subsets identified by mass cytometry. A significant positive association was found between IFNγ^+^ CD4^+^ T cells and the Th1-subset identified by mass cytometry, while a negative but non-significant association was observed between IFNγ^+^ CD4 T cells and Th2 cells (Fig. 6b). These data suggest that the Th1/Th2-phenotyping based on mass cytometry surface markers, corresponded with the production of the Th1 hallmark cytokine IFNγ. Despite the statistical significance, it is worth noting that these correlations were relatively weak, indicating the complex nature of phenotype-function associations.

**Fig. 7:**
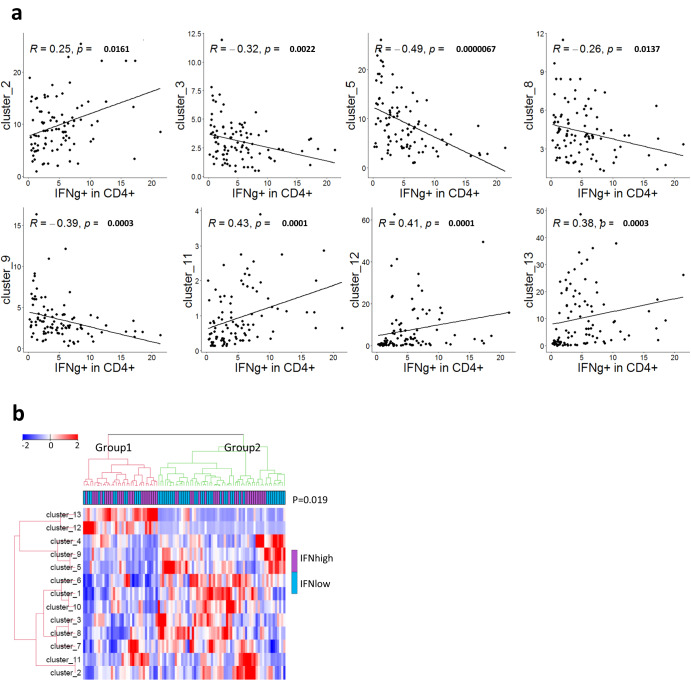
IFNγ production correlates with CD4^+^ T cell clusters identified by unsupervised analysis. PBMCs from BC (*n* = 95) patients were stimulated by CD3/CD28 beads and IFNγ production was determined by flow cytometry. **a** Spearman rank correlation of CD4^+^ T cell clusters (shown in Fig. 4) with IFNγ production by CD4^+^ T cells. Only T cell clusters with a significant correlation are shown. FDR-adjusted p-values are shown. **b** Heat map (hierarchical clustering) representing relative abundance of the 13 CD4^+^ T clusters in IFN^high^ and IFN^low^ BC patients. Groups were compared with Fisher’s exact test.

In addition, we investigated how the IFNγ production correlated with the CD4^+^ T cell clusters identified by unsupervised analysis. Clusters 2, 11, 12, 13 associated positively with IFNγ production, while cluster 3,5, 8, 9 associated negatively (Fig. 7a). Interestingly, cluster 2 and 11 were CXCR3^+^ and negative for the majority of the immune checkpoint receptors. Whereas cluster 5 and 9 were naïve T cell clusters, cluster 8 was Tregs, and cluster 3 co-expressed multiple immune checkpoints. Next, we divided the patients into IFNγ^high^ and IFNγ^low^ producers based on the median. Hierarchical clustering and PART-guided classification of BC patients led to two subgroups of patients. BC-group1 had a higher proportion of IFNγ^high^ producers (*p* = 0.019; Fig. 7b) and was particularly enriched for cluster 12 and 13 that were positive for granzyme-B and negative for most of the immune checkpoints.

### Association of peripheral immune profile with Immune cell infiltration in tumor

**Fig. 8:**
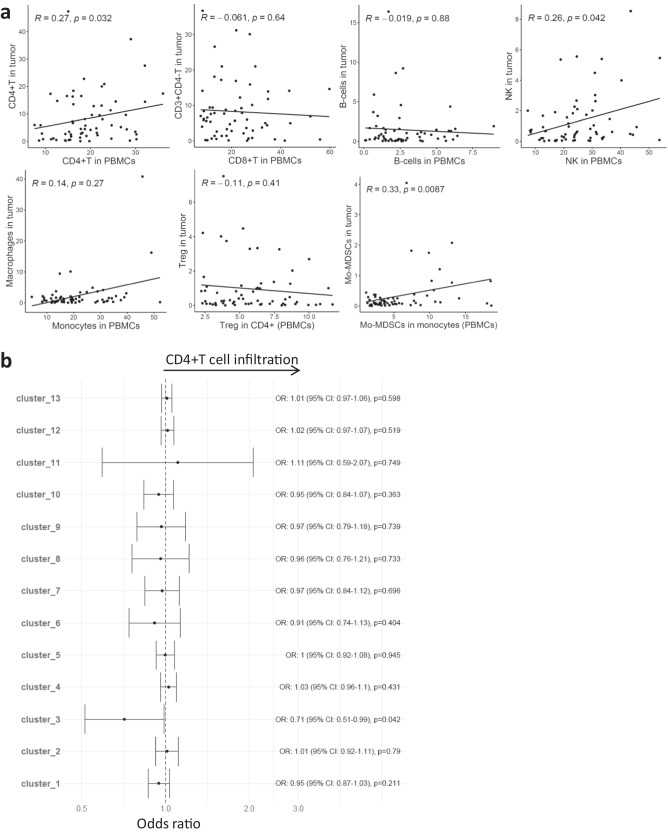
Association of immune cell types in PBMCs with immune cells infiltration in tumor. **a** Correlation of immune cells between peripheral blood and tumor. Spearman correlation. **b** Odds ratios (OR) for associations between CD4^+^ T cell clusters in PBMCs and CD4^+^ T cell infiltration in tumor. Odds ratios were derived from univariate logistic regression. Immune cells in tumor were shown as their proportion within live cells as determined by flow cytometry. Unadjusted *p*-values are shown. BC (*n* = 63).

To investigate how the immune cell profile in blood related to the immune cell composition in tumor, we analyzed tumor biopsies from 63 of the 104 mBC patients, where we obtained fresh tumor that was dissociated into single cells. This analysis was performed on all biopsies where there was sufficient material. Patient characteristics for the 63 patients are shown in Supplementary Table 2. The tumor biopsies were obtained at the same time point (within 0–3 weeks) as the PBMCs, and the patients received no treatment in-between these samplings. By flow cytometry, we determined the abundance of different immune cell types (CD4^+^ T cells, CD4^-^ T cells, B-cells, NK cells, Macrophages, Tregs, Mo-MDSCs ; gating strategy Supplementary Fig. 11) in tumor. Several moderate, but significant (unadjusted *p* < 0.05), associations were observed between immune cell proportions in tumor and blood. The CD4^+^ T and NK cell proportions in PBMCs correlated with CD4^+^ T and NK cells in the tumors (Fig. 8a). Moreover, the Mo-MDSCs proportions in blood monocytes positively correlated with Mo-MDSCs in tumor (Fig. 8a). Observing a correlation between CD4^+^ T cells in blood and tumor, we asked if specific CD4^+^ T phenotypes could predict T cell infiltration into tumor. To this end, we categorized tumors as CD4^high^ or CD4^low^ (=<median of % CD4^+^ T cells in tumor) and fit logistic regression models to predict T cell infiltration by the CD4^+^ T cell clusters (in PBMCs) that we had identified by unsupervised CyTOF analysis (Fig. 4). We found that the proportion of cluster 3 in blood was negatively associated with CD4^+^ T cell infiltration in tumor (OR = 0.71, 95%CI = 0.51-0.99, *p* < 0.05; not significant after FDR correction) (Fig. 8b). A similar negative association was seen with overall CD3^+^ T cell infiltration in tumor (OR = 0.72, 95%CI = 0.52-1.0, *p* = 0.05). Interestingly, cluster 3 co-expressed multiple immune checkpoint receptors and negatively correlated with IFN-γ production (Fig. 7a).

### Association of peripheral immune subsets with response to therapy

We further investigated if the observed peripheral immune cell populations were associated with clinical benefit in the randomized, placebo-controlled ALICE trial^22^, which evaluated the addition of atezolizumab (anti-PD-L1) to immune-stimulating chemotherapy. In this trial, 68 patients received pegylated liposomal doxorubicin (PLD) and low-dose cyclophosphamide in combination with atezolizumab (atezo-chemo) or placebo (placebo-chemo). The trial showed improved clinical outcome for patients in the atezolizumab-chemotherapy arm. CYTOF data on PBMCs were available from 29/40 patients in the atezo-chemo arm, and 18/28 patients in the placebo-chemo arm. In the ALICE protocol, clinical benefit was defined as either an objective tumor response by iRECIST^23^ or stable disease lasting at least until the radiological evaluation at 24 weeks ± 7 days. We applied the same definition of clinical benefit in the present analysis.

**Fig. 9:**
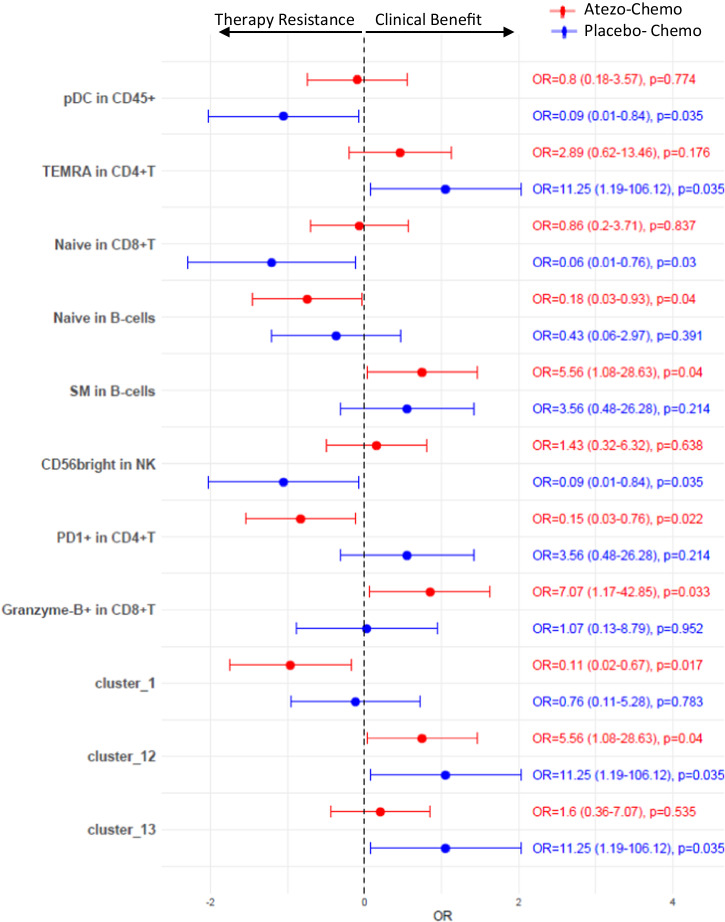
Association of peripheral immune cells with clinical benefit in mTNBC patients treated with atezolizumab with/without anthracycline-based chemotherapy. Forest plot showing odds ratios (OR). Clinical benefit was defined as patients who had either an objective response or stable disease lasting at least until the radiological evaluation at 24 weeks ± 7 days. Odds ratios were derived from univariate logistic regression. Unadjusted *p*-values are shown. Only factors with *p*-values < 0.05 in one or both treatment arms are shown. Atezo-Chemo; patients receiving combined Atezolizumab plus anthracycline-based chemotherapy (*n* = 29), Placebo-Chemo; patients receiving anthracycline-based chemotherapy only (*n* = 18).

Logistic regression analysis showed that none of the major immune cell subsets (shown in Fig. 1) were significantly associated with clinical benefit except pDCs, which were associated with poor outcome in the placebo-chemo arm (OR = 0.09, *p* = 0.034) (Fig. 9, Supplementary Table 3). Among the major differentiation subsets of T cells, CD4^+^ TEMRA T cells were positively associated with clinical benefit (OR = 11.25, *p* = 0.035) in placebo-chemo arm, while CD8^+^ Naive T cells (OR = 0.06, *p* = 0.03) and CD56^bright^ NK cells (OR = 0.09, *p* = 0.035) were associated with treatment resistance (Fig. 9, Supplementary Table 3).

In the atezo-chemo arm, further analysis revealed that increased numbers of Granzyme-B^+^ CD8 T cells were positively associated with clinical benefit (OR = 7.07, *p* = 0.033), while increased numbers of PD1^+^ CD4 T cells were associated with poor outcome (OR = 0.15, *p* = 0.022) (Fig. 9, Supplementary Table 3). Further, increased proportions of a naive phenotype in B-cells were associated with inferior outcome (OR = 0.18, *p* = 0.04), while higher proportions of SM B-cells were associated with better outcome (OR = 5.56, *p* = 0.04; Fig. 9, Supplementary Table 3).

Analysis of the 13 CD4^+^ T cell clusters identified above (Fig. 4) revealed that cluster 1 (CCR4^+^CXCR3^-^ CD4) was associated with poor clinical outcome in the atezo-chemo arm (OR = 0.11, *p* = 0.017; Fig. 9, Supplementary Table 3), while cluster 12 (PD1^-^Granzyme-B^+^ CD4) was positively associated with clinical outcome in both treatment arms (atezo-chemo arm OR = 5.56, *p* = 0.04; placebo-chemo arm OR = 11.25, *p* = 0.035) (Fig. 9, Supplementary Table 3). Cluster 13 (PD1^+^Granzyme-B^+^ CD4) was positively associated with clinical outcome only in placebo-chemo arm (OR = 11.25, *p* = 0.035; Fig. 9, Supplementary Table 3). We also noted that cluster 3 (CD4 T cells co-expressing multiple immune checkpoints), which we have shown to be associated with reduced IFNγ production and less T cell infiltration in tumors, appeared moderately associated with poor outcome in both arms, but with non-significant p-values (atezo-chemo arm OR = 0.53, *p* = 0.413; placebo-chemo arm OR = 0.29, *p* = 0.324) (Supplementary Table 3). None of these factors were significant after FDR correction. The OR with 95% CI and p-values for all factors tested are shown in Supplementary Table 3.

## Discussion

In the current study, we characterized the phenotype of peripheral blood immune cells in metastatic BC patients and compared it to age-matched HD. We also determined whether phenotype corresponded to T cell functionality and if it was related to molecular BC subtypes or previous therapies. The results show that the composition of PBMCs in metastatic BC patients is heavily skewed, with a significant reduction in CD4^+^ T cells and an increase in monocytes. Moreover, there was a numerical reduction in B cells and an increase in NK cells as a proportion of leukocytes, though these differences were not statistically significant. We further observed increased levels of Tregs, as well as altered phenotypes of effector T cells, Tregs and B-cells. The findings suggest an immune-suppressed environment and are consistent with a chronic inflammation signature. We also found that the HR^+^ BC and TNBC patients in general had similar PBMC profiles, and that the differences appeared mainly related to previous therapies, rather than to BC subtype. The fact that nearly all untreated patients belonged to the TNBC group could be a confounding factor, but most TNBC patients had received previous treatments, and their profile resembled the HR^+^ BC group.

Leukocyte depletion is a hallmark of chemotherapy. However, little is known about the durability of lymphocyte depletion in BC patients and the effect of chemotherapy on lymphocyte subsets and phenotype. A study from Verma et al. in patients with primary BC indicated that B-cells and CD4^+^ T cells remain affected for some time after adjuvant chemotherapy^24^. There is also limited information on the effect of CDKi on immune cell populations except for neutrophils. One recent study found no significant changes in T cell frequency after CDKi treatment, but a decrease in Treg and MDSC percentages^25^. In our cohorts of metastatic TNBC and HR^+^ BC, we found that the general lymphocyte count was significantly reduced for patients treated with chemotherapy or CDKi, even if such treatment had not been given in the last 3 months. Others have found that CD8^+^ T cell numbers return quickly to baseline after chemotherapy is stopped, while CD4^+^ T cells remain decreased, with an expansion of the effector memory pool^24,26,27^. We observed that both chemotherapy- and CDKi- treated patients displayed an increase in memory CD4^+^ T cells and a reduction of naive CD4^+^ T cells. Furthermore, we found that treated patients had increased numbers of naive B cells and a decreased number of memory B cells. These observations are in line with the findings from the adjuvant chemotherapy regime studied by Verma and collegues^24^, whereas similar data are to our knowledge not available for CDKi. Mature B cells can generate tumor-specific antibodies and promote T-cell activation via the presentation of tumor antigens to CD4^+^ T cells^28,29^. The predominantly naive phenotype of B cells in BC patients may therefore lead to less potent adaptive immune responses.

In the myeloid compartment, we found that the BC patients had reduced levels of CD16^−^ mDCs, which are potent antigen-presenting cells, but also less M2 monocytes. It should be underlined that these observations do not necessarily reflect the frequency or phenotype of DCs and macrophages in lymph nodes or tumor. High lymphocyte-monocyte ratios in peripheral blood have been shown to be associated with better prognosis in BC patients^7,12^, presumably consistent with anti-tumoral response of T/B cells and pro-tumorigenic roles of subsets of monocytes^21^. In our data set, the phenotype of monocytes was not very different between BC patients and HD. However, we observed an increased ratio of MDSCs to T cells, which could suppress the immune response.

The repeated exposure of tumor antigens could over time lead to chronic inflammation, which may give a pro-tumor microenvironment and enable immune escape through the expression of immune checkpoints, increase in certain cytokines and the generation of Tregs and other immuno-suppressive cells. We found the increased expression of immune checkpoint receptors and an enrichment of EM-T cells, Tregs and effector cells with a Th2 or Th17 phenotype, along with a dysregulated monocytes/T-cells ratio. This profile resembles an immune signature elicited by chronic inflammation. Although during the early inflammatory phase multiple checkpoints are induced on T cells, sustained chronic inflammation leads to terminal exhaustion. Several pro-inflammatory cytokines such as IL-6 can promote T cell exhaustion through IL-6/STAT3/PD-1 transcription regulation^30^. In melanoma, VEGF production by malignant cells is reported to induce Th2-mediated chronic inflammation^31^. Several studies have shown high serum concentration of VEGF in BC patients^32^.

There are only a few reports on the expression of immune checkpoint receptors on peripheral blood cells from BC patients compared to healthy individuals. Elashi et al. found increased gene expression of PD-1, CTLA-4, TIM-3, TIGIT, and PDL-1 in the peripheral blood of primary BC patients, while LAG-3 expression was downregulated^33^. Another study by Liu et al. found increased proportion of PD1^+^ CD8 T cells in peripheral blood of BC patients, however other immune checkpoint receptors were not investigated^34^. We demonstrated increased expression, at the protein level, of various immune checkpoint molecules such as PD1, TIGIT, TIM3 and ICOS. These molecules could indicate both transiently activated and exhausted T cell states. Several studies have suggested, however, that while the induction of individual checkpoint molecules can reflect transient activation, the co-expression of multiple checkpoint molecules indicates T cell exhaustion^35–38^. In our study, we identified a CD4^+^ cell cluster (cluster3) that co-expressed multiple immune checkpoint receptors and was negatively associated with cytokine production by T cells in functional assays. In most cancer forms, including TNBC and HR^+^ BC^39–43^, many patients with the expression of PD-L1 in the tumor tissue do not respond to PD1/PD-L1 blockers. This observation may point to other mechanisms of immune escape, including the co-expression of multiple checkpoint molecules, consistent with T cell exhaustion.

Tregs suppress immune responses and promote a pro-tumor environment^44–46^. Our finding of increased Tregs in patients, compared to HD, is consistent with studies in a variety of cancers including BC^47^. Moreover, we found that increased Treg numbers was negatively associated with T cell cytokine production. This observation supports the functional relevance of the mass cytometry-based phenotyping. Interestingly, the Tregs expressed increased levels of PD1, TIGIT and TIM3. While these checkpoint receptors may mark exhaustion in conventional T cells, they serve a different role on Tregs and are critically important for their suppressive function^48–50^. Furthermore, CD39 and ICOS were highly expressed on Tregs in our patient cohort. These molecules indicate highly suppressive Tregs^51–53^. The results thus suggest an increased suppressive capacity of Tregs in the BC patients, both as assessed by numbers and phenotype.

There is sparse information on treatment-induced changes of checkpoint inhibitor expression in BC patients. In our dataset, several immune checkpoints were highly expressed on T cells from treated patients. Untreated patients also showed higher expression of TIGIT, TIM3, and ICOS compared to HDs, but the difference did not reach statistical significance. The untreated patients in our cohort represented a small group. We still noted that their immune profiles generally fell between HD and treated patients, suggesting an immune cell dysregulation in BC patients, becoming more pronounced after treatment. Whether the changes are due to disease or treatment, the observed immune profile represent a relevant milieu for immunotherapies against advanced BC.

Effector T cells are conventionally classified into Th1, Th2, Th17 and other subsets, based on their cytokine profile, which influences their ability to mount effective anti-tumor responses. Many studies have shown opposing roles for Th1 and Th2 CD4^+^ T cells as being anti-tumorigenic and pro-tumorigenic respectively^54^. However, there have been contrasting reports on the role of Th2 cells in BC, indicating that their role may be context dependent. Several studies have shown that BC patients with Th2 dominated cytokine profiles have a poor prognosis^55–57^. In line with this, pharmaceutical targeting of Th2 cells enhances immunotherapy response in murine BC models^58^. On the other hand, a recent study indicated that Th2 cells suppress BC by inducing terminal differentiation of tumor cells^59^. Further, another interesting study suggests that the Th2-cytokine IL-5 mediates increased response to immune checkpoint inhibitors in BC through the recruitment of eosinophils^60^. High numbers of Th17 cells have been reported in variety of solid cancers and have been shown to be associated with a poor prognosis^61^. In our cohort of BC patients, we observed higher numbers of Th2 and Th17 CD4^+^ T cells, while the Th1 CD4^+^ T cells were largely comparable between HD and BC. This skewed Th1/Th2 ratio, in combination with a generally suppressed immune environment, could generate a suboptimal anti-tumor immune response.

It is important to assess how the identified immune phenotypes correlate with functional T cell responses. We found that IFNγ production correlated with the presence of more differentiated T cells (EM and TEMRA), while inversely correlating with Tregs. Furthermore, we identified that several CD4^+^ T cells clusters expressing CXCR3^+^ (Cluster 2 and 11) or Granzyme-B^+^ (cluster 12 and 13) associated positively with IFNγ production, whereas Cluster 3, which expressed multiple immune checkpoints, and Cluster 8 (Tregs) associated negatively with IFNγ production. The observed correlations between phenotype and IFNγ production were mostly weak. However, the findings were in line with current immunological understanding and suggest that mass cytometry identified phenotypes can be indicative of immune function. Others have reported that co-expression of multiple checkpoints is associated with terminal T cell dysfunction^62,63^ and an unfavorable outcome in a variety of cancer forms. An interesting study indicated that co-inhibition of PD1, TIGIT, and TIM3 led to the reactivation of tumor specific T cells^64^.

We further investigated how the peripheral immune cell profile related to immune cells in tumor. The results showed that the proportions of CD4^+^ T cells, NK cells and Mo-MDSCs in blood weakly correlated with their counterparts in tumor. Further, one of the CD4^+^ T cell clusters was negatively associated with CD4^+^ T cell infiltration in the tumor. Interestingly, this T cell cluster co-expressed multiple immune checkpoints and negatively correlated with IFNγ production in the functional T cell assays. This finding requires validation in an independent cohort, but may indicate a link between an immunosuppressed exhausted T cell state in the periphery with low T cell infiltration into the tumor.

Furthermore, it is important to understand how peripheral immune cell features influence the effect of immunotherapy, and if these features may be informative as biomarkers for therapeutic benefit. We explored this by investigating the association of the observed immune cell features with benefit from therapy in the ALICE trial. The results showed that increased proportions of Granzyme-B^+^ CD8 T-cells and SM B cells were associated with clinical benefit in the atezo-chemo arm, but not in the chemo-placebo arm. These observations should be interpreted with caution, as multiple comparisons were made, but may reflect that patients with an ongoing immune response are more likely to respond to PD-L1 blockers. We currently conduct further analyses of PBMCs and biopsies obtained at baseline and multiple on-treatment time-points in the ALICE and ICON trials. It will be of interest to see how the study treatments affected the immune milieu and if on-treatment changes could be predictive of response.

In conclusion, we observed immune signatures in BC patients suggestive of chronic inflammation, with an upregulation of suppressive mechanisms comprising both regulatory cells and immune checkpoints. The observed profile is not necessarily a consequence of metastatic disease, as it may be influenced by previous therapies and could reflect phenotypes related to treatment resistance. There is also most likely an immune-mediated selection of patients developing metastatic disease, and the observed immunosuppressive features may have been present at disease onset. The interpretation of treatment related effects is limited by the fact that patients were only analyzed at a single time-point. The main purpose of this study, however, was not to identify the causes of any immune dysregulation, but to provide insight into the systemic immune status of patients that are candidates for immunotherapy. The patients in the present study had advanced disease and were all screened for participation in experimental trials investigating checkpoint inhibitor therapy. These results indicate that several immuno-suppressive mechanisms are upregulated in metastatic BC and may be considered as therapeutic targets. PBMC profiles may be obtained at repeated time points for use as biomarkers, circumventing the need for invasive procedures. It should still be emphasized, that the observations from peripheral blood do not necessarily reflect the milieu in lymph nodes or the tumor microenvironment. Further studies may focus on how the observed PBMC signatures differ among BC patients, if they change during therapy and further explore if the signatures are predictive of clinical response to immunotherapy or other treatment modalities.

## Methods

### Study subjects

A total of 104 patients with metastatic HER2^−^ BC (mean age = 54 years, range = 29–75 years, female = 103) and 20 age- and gender-matched healthy donors (HD; mean age = 50.5 years, range = 42–71 years, all females) were included in the study from Oslo University Hospital (OUH), Stavanger University Hospital, and St. Olavs Hospital, Norway. Material from the BC patients and HD was obtained as part of two clinical trials (ALICE NCT03164993^22,65^; ICON NCT03409198^66^) approved by the Norwegian Medicines Agency, Institutional Review Board and Regional Committees for Medical Research Ethics (2016/1750, 2017/1283). Written informed consent was obtained from all patients. The HD were anonymized. The trials were conducted in compliance with the World Medical Association Declaration of Helsinki.

### PBMCs isolation and mass cytometry staining

PBMCs from BC patients and HD were isolated using LymphoPrep density-gradient centrifugation (Abbott Rapid Diagnostics, Norway), and cryopreserved in liquid nitrogen until use. Frozen PBMCs were thawed at 37 °C and processed for mass cytometry. An internal PBMCs control was included in each run to minimize experimental variation. In brief, ~3 × 10^6^ PBMCs from each patient/donor were subjected to a 5-choose-2 barcoding scheme using combinations of CD45 antibodies (RRID:AB_314390, Biolegend, CA, USA) to ensure that each sample stains positive for 2 of the available 5 barcode channels. Further staining was performed as per manufacturer´s recommendations (Standard BioTools, CA, USA), and stained cells were then resuspended in Cell Acquisition Solution-EQ Bead mixture (Standard BioTools) to a concentration of 5 × 10^5^ cells/mL. The antibodies were obtained from Standard BioTools or conjugated in-house using Maxpar X8 Ab labeling kits (Standard BioTools) (Supplementary Table 4).

### Mass cytometry and data acquisition

The stained barcoded samples were resuspened in Cell Acquisition Solution (CAS) and loaded onto a Helios CyTOF system (Standard BioTools) using an attached autosampler with wide-bore injector and were acquired at a rate of 500 events/sec. Data were collected as .fcs files using CyTOF software (Version 6.7.1014, Standard BioTools). After acquisition, intra-file signal drift was normalized to the acquired calibration bead signal and individual files were deconvoluted and stored into .fcs files using FlowJo (version 10.8.1, FlowJo, OR, USA). File clean-up (e.g., removal of dead cells, debris, doublets, and beads) was performed using FlowJo. In total, 124 samples were run on a Helios CyTOF system spanning 13 runs.

### Mass cytometry data analysis

The data files from several runs were batch corrected using cyCombine^67^. The data were either analyzed through manual gating (FlowJo) or subjected to dimensionality reduction and automated clustering using UMAP and PhenoGraph (Cytofkit2)^68^. All downstream analysis was done in R version 4.1.2. Two groups were compared by Wilcoxon Mann–Whitney Rank Sum Test. More than two groups were compared by Kruskal–Wallis test, followed by pair-wise comparisons using Wilcoxon Mann–Whitney Rank Sum Test. Correlation analysis was done using spearman correlation. Multiple testing correction (Benjamini-Hochberg FDR) was applied separately to the major immune subsets (Fig. 1) and to the phenotypic subsets within each cell type (i.e, proportions within CD4^+^ T, CD8^+^ T, B-cells, monocytes, NK, mDC, and Tregs). FDR-corrected two-sided *p*-values (*p*.adj) are shown throughout the manuscript unless explicitly stated otherwise in the text or figure legends. *P*.adj-values < 0.05 were considered statistically significant. Exact p-values (nominal and FDR-adjusted) are reported in the Supplementary Tables 5–7. The center line in box plots represents the median value and the box limits represent the upper (75 percentile) and lower quartiles (25 percentile). The whiskers extend to the extreme values, omitting outliers extending >1.5 x IQR from the box limits, outliers are shown as dots.

### Flow cytometric analysis of PBMCs

5×10^5^ PBMCs from each patient/donor were stained with antibodies CD3-BUV395 (RRID:AB_2744382), CD4-BV510 (RRID:AB_2869877) (BD Biosciences, NJ, USA), CD25-BV605 (RRID:AB_2800963, BioLegend) and Fixable Viability Dye eFluor780 (ThermoFisher). After fixation and permeabilization using Foxp3 /Transcription Factor Staining Buffer Set (ThermoFisher), PBMCs were incubated with antibody Foxp3-PE (RRID:AB_1944444, ThermoFisher). Samples were acquired using BD FACSymphony A5 flow cytometer (BD Biosciences), and the data analyzed with FlowJo.

### Cytokine analysis

3×10^5^ PBMCs were plated into each well of a 96 well round-bottomed plate and stimulated for 1 h with CD3/CD28 T-cell activator beads (2:1 T-cell:bead ratio). After 1 h, brefeldin A (10 µg/mL) was added and the cells incubated for a further 15 h. PBMCs were surface stained with the antibodies CD3-BUV395, CD4-BV510 (BD Biosciences), CD8-BV711 (RRID:AB_2565242, Biolegend) and Fixable Viability Dye eFluor780 (ThermoFisher). After fixation and permeabilization using CytoFix /CytoPerm Buffer (BD Biosciences), PBMCs were incubated with antibody IFNγ-FITC (RRID:AB_465415, ThermoFisher). Samples were acquired using BD FACSymphony A5 flow cytometer (BD Biosciences), and the data analyzed with FlowJo.

### Flow cytometric analysis of tumor biopsies

Out of the 104 mBC patients included in the mass cytometry analysis, tumor biopsies were obtained from 63 patients (HR^+^ BC, *n* = 42; TNBC, *n* = 21) and were subjected to flow cytometry. Briefly, freshly obtained tumor biopsies were mechanically chopped and then dissociated in DMEM F12 medium containing 10% FBS, collagenase-IV (1 mg/mL), hyaluronidase (1 mg/mL), & DNase-I (50 µg/mL) in a 37 °C water bath for 45 min. Single cell suspensions were then filtered, washed with PBS, and stained with antibodies CD45-A488, CD3-Pacific blue, CD4-PE, CD25-BV605, CD14-PE-Cy7, CD33-BV711, HLA-DR-BV650 (Biolegend), CD127-BV786 (BD Biosciences), and Fixable Viability Dye (eFluor780, ThermoFisher). Samples were acquired using LSR-II/FACSymphony A5 flow cytometer (BD Biosciences), and the data analyzed with FlowJo.

### Supplementary information

Supplementary data

Supplementary Table 3

Supplementary Table 5

Supplementary Table 6

Supplementary Table 7

reporting-summary

## Data Availability

Data generated from this study will be available upon reasonable request. The data is subject to patient confidentiality, and the transfer of data will require approval from the Regional Committee for Medical and Health Research Ethics South-East Norway. Requests should be made to the corresponding author (jonky@ous-hf.no).

## Code availability

Data analysis and data presentation were performed using open-source R (version 4.1.2) packages. R codes are available from the corresponding author upon reasonable request.
