# Supplementary material for: Peripheral immune cells in metastatic breast cancer patients display a systemic immunosuppressed signature consistent with chronic inflammation
Source: NPJ Breast Cancer. 2024 Apr 23;10:30. doi: 10.1038/s41523-024-00638-2 (PMC11039627; doi:10.1038/s41523-024-00638-2)
Supplement: Supplementary file 1 — Supplementary data [file 41523_2024_638_MOESM1_ESM.pdf]

Peripheral immune cells in metastatic breast cancer patients display a systemic immunosuppressed signature consistent with chronic inflammation

SUPPLEMENTARY DATA

Supplementary Table 1 – Characteristics of breast cancer patients grouped based on previous treatments

	**Chemo-CDK-**	**Previous Chemo/CDK (not last 3 months)**	**Chemo (last 3 months)**	**CDK (last 3 months)**
	**n=10**	**n=40**	**n=26**	**n=28**
Age (years)	57.5 (38.0-69.0)	55.0 (48.0-61.5)	53.0 (44.0-59.0)	53.0 (50.0-62.5)
Sex				
Male	0 (0.0%)	1 (2.5%)	0 (0.0%)	0 (0.0%)
Female	10 (100.0%)	39 (97.5%)	26 (100.0%)	28 (100.0%)
Breast cancer subtype				
Triple negative BC (TNBC)	9 (90.0%)	22 (55.0%)	16 (61.5%)	0 (0.0%)
HR+/HER2- BC (HR+BC)	1 (10.0%)	18 (45.0%)	10 (38.5%)	28 (100.0%)
Denovo metastatic disease	9 (90.0%)	3 (7.5%)	5 (19.2%)	8 (28.6%)
Disease stage at sampling				
Stage IV	10 (100.0%)	40 (100.0%)	26 (100.0%)	28 (100.0%)
Previous (neo)adjuvant chemotherapy	0 (0.0%)	37 (92.5%)	20 (76.9%)	17 (60.7%)
Previous Lines of metastatic chemotherapy				
None	10 (100.0%)	31 (77.5%)	0 (0.0%)	15 (53.6%)
1 line	0 (0.0%)	7 (17.5%)	26 (100.0%)	13 (46.4%)
2 lines	0 (0.0%)	2 (5.0%)	0 (0.0%)	0 (0.0%)
Chemotherapy naive	10 (100.0%)	0 (0.0%)	0 (0.0%)	7 (25.0%)
Previous CDK4/6 inhibitor	0 (0.0%)	16 (40.0%)	11 (42.3%)	28 (100.0%)
Previous adjuvant radiotherapy	0 (0.0%)	31 (77.5%)	20 (76.9%)	23 (82.1%)
Previous palliative radiotherapy	0 (0.0%)	8 (20.0%)	10 (38.5%)	12 (42.9%)
Initial diagnosis to sampling (months)	1.1 (0.9-1.5)	63.4 (30.6-112.2)	80.2 (32.9-120.6)	88.3 (38.0-144.9)
Advanced diagnosis to sampling (months)	0.7 (0.2-1.1)	3.5 (1.0-24.7)	19.9 (7.2-43.1)	24.2 (16.4-33.3)
Bone metastases	2 (20.0%)	28 (70.0%)	15 (57.7%)	27 (96.4%)
Liver metastases	3 (30.0%)	23 (57.5%)	14 (53.8%)	21 (75.0%)
Lung metastases	4 (40.0%)	16 (40.0%)	7 (26.9%)	7 (25.0%)
More than 3 sites of metastases	0 (0.0%)	9 (22.5%)	7 (26.9%)	6 (21.4%)

Data are presented as median (IQR) for continuous measures, and n (%) for categorical measures.

Supplementary Table 2 – Characteristics of breast cancer patients included in flow cytometric analysis of tumors

	**All (n=63)**	**TNBC (n=21)**	**HR+BC (n=42)**
Age (years)	53.0 (49.0-62.0)	55.0 (43.0-59.0)	52.5 (49.0-62.0)
Sex			
Male	1 (2%)	0 (0%)	1 (2%)
Female	62 (98%)	21 (100%)	41 (98%)
Breast cancer subtype			
Triple negative BC (TNBC)	21 (33%)		
HR+/HER2- BC (HR+BC)	42 (67%)		
Denovo metastatic disease	14 (22%)	5 (24%)	9 (21%)
Previous (neo)adjuvant chemotherapy	46 (73%)	16 (76%)	30 (71%)
Previous Lines of metastatic chemotherapy			
None	37 (59%)	15 (71%)	22 (52%)
1 line	25 (40%)	5 (24%)	20 (48%)
2 lines	1 (2%)	1 (5%)	0 (0%)
Chemotherapy naive	11 (17%)	4 (19%)	7 (17%)
Previous CDK4/6 inhibitor	41 (65%)	1 (5%)	40 (95%)
Previous adjuvant radiotherapy	45 (71%)	12 (57%)	33 (79%)
Previous palliative radiotherapy	21 (33%)	3 (14%)	18 (43%)
Initial diagnosis to sampling (months)	73.4 (29.9-122.9)	29.9 (21.5-88.6)	83.3 (44.4-145.0)
Advanced diagnosis to sampling (months)	18.7 (3.0-32.8)	1.9 (0.7-6.2)	25.4 (17.5-37.5)
Bone metastases	48 (76%)	8 (38%)	40 (95%)
Liver metastases	38 (60%)	5 (24%)	33 (79%)
Lung metastases	19 (30%)	10 (48%)	9 (21%)
More than 3 sites of metastases	15 (24%)	2 (10%)	13 (31%)

Data are presented as median (IQR) for continuous measures, and n (%) for categorical measures.

Supplementary Table 3 - Association of peripheral blood immune cells with clinical benefit in ALICE trial (provided as excel file)

Supplementary Table 4 – Mass cytometry panel used in the study

**Antibody/Marker**	**Metal Tag**	**Clone**	**Manufacturer**
CD45 (Barcode)	89Y	HI30	Standard BioTools, CA, USA
CD45 (Barcode)	104Pd	HI30	Biolegend, CA, USA
CD45 (Barcode)	106Pd	HI30	Biolegend, CA, USA
CD45 (Barcode)	108Pd	HI30	Biolegend, CA, USA
CD45 (Barcode)	110Pd	HI30	Biolegend, CA, USA
CD45 (Barcode)	In-115	HI30	Biolegend, CA, USA
CCR6	141Pr	G034E3	Standard BioTools, CA, USA
CD19	142^{Nd}	HIB19	Standard BioTools, CA, USA
CD45RA	143Nd	HI100	Standard BioTools, CA, USA
CD38	144Nd	HIT2	Standard BioTools, CA, USA
CD163	145Nd	GHI/61	Standard BioTools, CA, USA
IgD	146Nd	IA6-2	Standard BioTools, CA, USA
CD303 (BDCA2)	147Sm	201A	Standard BioTools, CA, USA
CD14	148Nd	RMO52	Standard BioTools, CA, USA
CD25	149Sm	2A3	Standard BioTools, CA, USA
KIR2D	150Nd	NKVFS1	Miltenyi Biotec, Germany
KIR3DL1/2	150Nd	5.133	Miltenyi Biotec, Germany
ICOS	151Eu	C398.4A	Standard BioTools, CA, USA
TCRgd	152Sm	11F2	Standard BioTools, CA, USA
TIM3	153Gd	F38-2E2	Standard BioTools, CA, USA
TIGIT	154Eu	MBSA43	Standard BioTools, CA, USA
PD1	155Gd	EH12.2H7	Standard BioTools, CA, USA
CXCR3	156Gd	G025H7	Standard BioTools, CA, USA
CCR4	158Eu	205410	Standard BioTools, CA, USA
CCR7	159Tb	G043H7	Standard BioTools, CA, USA
CD39	160Gd	A1	Standard BioTools, CA, USA
Ki67	161Dy	B56	Standard BioTools, CA, USA
CD11c	162Dy	Bu15	Standard BioTools, CA, USA
CD33	163Dy	WM53	Standard BioTools, CA, USA
CD161	164Dy	HP-3G10	Standard BioTools, CA, USA
CD127	165Ho	A019D5	Standard BioTools, CA, USA
CD15s	166Er	CSLEX1	BD biosciences
CD27	167Er	O323	Standard BioTools, CA, USA
CD8	168Er	SK1	Standard BioTools, CA, USA
NKG2A	169Tm	Z199	Standard BioTools, CA, USA
CD3	170Er	UCHT1	Standard BioTools, CA, USA
Granzyme-B	171Yb	GB11	Standard BioTools, CA, USA
CD57	172Yb	HCD57	Standard BioTools, CA, USA
HLA-DR	173Yb	L243	Standard BioTools, CA, USA
CD4	174Yb	SK3	Standard BioTools, CA, USA
PDL1	175Lu	29E.2A3	Standard BioTools, CA, USA
CD56	176Yb	N901	Standard BioTools, CA, USA
CD16	209Bi	3G8	Standard BioTools, CA, USA
Intercalator-Ir	191/193	-	Standard BioTools, CA, USA
Cisplatin-194Pt	194	-	Standard BioTools, CA, USA

Supplementary Table 5 - Nominal and FDR-adjusted p-values reported in the manuscript (HD vs. BC patients comparison) (provided as excel file)

Supplementary Table 6 - Nominal and FDR-adjusted p-values reported in the manuscript (HR+BC vs. TNBC comparison) (provided as excel file)

Supplementary Table 7 - Nominal and FDR-adjusted p-values reported in the manuscript (HD vs. Treatment groups comparison) (provided as excel file)

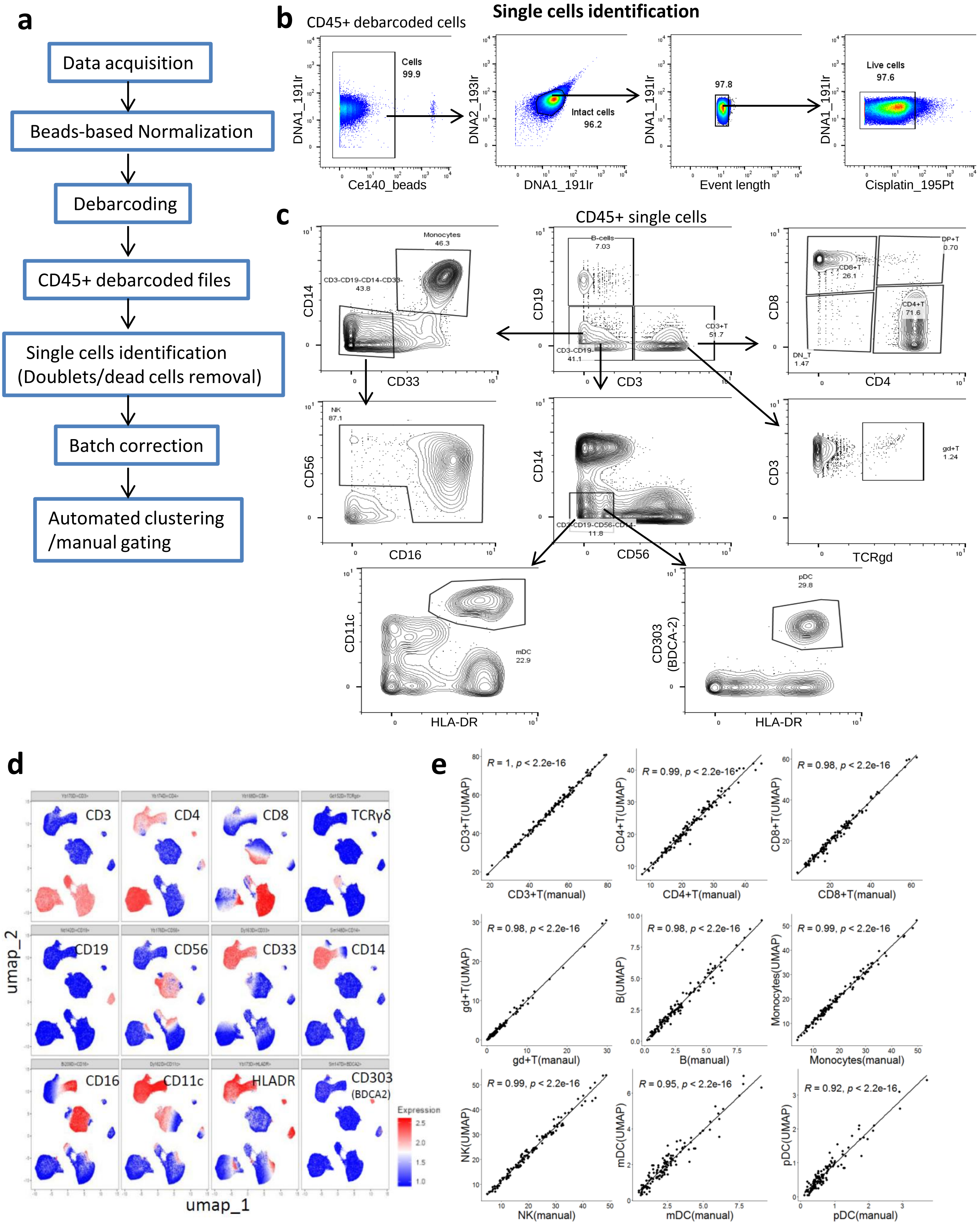

Supplementary Figure 1. (a) General scheme of pre-processing of CyTOF data. **(b)** Identification of single cells from debarcoded files. **(c)** Manual gating strategy used for identification of different immune subsets in PBMCs of BC patients and HD by CyTOF. **(d)** Expression of core phenotypic markers on UMAP. **(e)** Correlation between major immune subsets estimated by UMAP-phenograph guided approach and manual gating. Spearman rank correlation. p-value < 0.05 was considered statistically significant. *Abbreviations:* T, T cells; B, B cells; NK, Natural Killer cells; mDC, myeloid dendritic cells; pDC, plasmacytoid dendritic cells

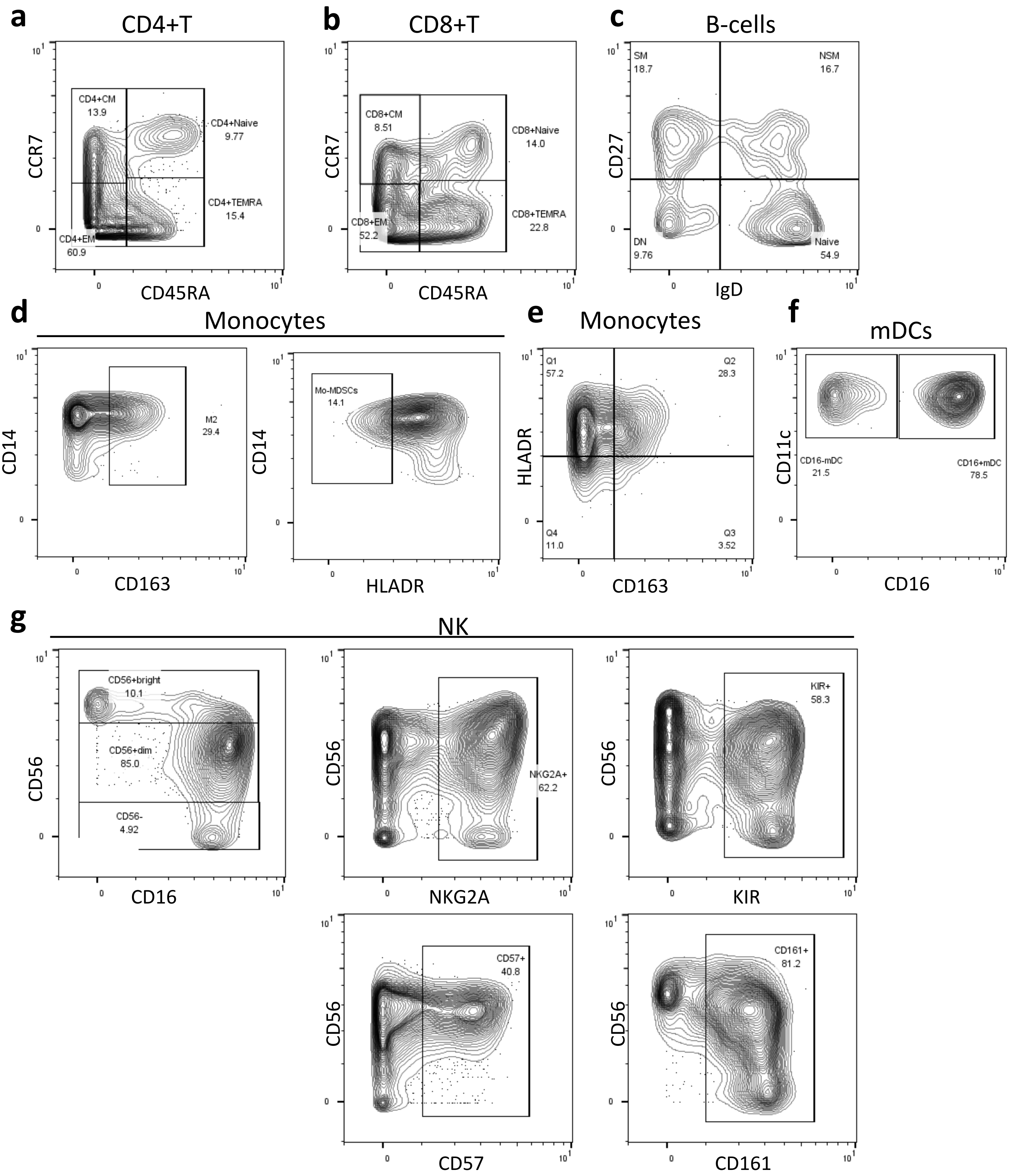

Supplementary Figure 2. Gating scheme for identifying the immune cell subsets corresponding to figure 2. **(a-b)** CD4 and CD8 T cells were divided into 4 subsets (Naive, CM, EM, and TEMRA) based on expression of CD45RA and CCR7. **(c)** B-cells were divided into 4 subsets (Naive, DN, SM, and NSM) based on expression of IgD and CD27. **(d)** Monocytes were classified as M2 (CD4+CD163+) and Mo-MDSCs (CD14+HLADR-). **(e)** FACS Plot showing co-expression of CD163 and HLADR on monocytes. **(f)** mDCs were grouped into 2 subsets (CD16- and CD16+ mDCs) based on CD16 expression. **(g)** NK cells were grouped into CD56bright, CD56dim and CD56- subsets based on CD56 and CD16 expression. Additionally NKG2A+, KIR+, CD57+ and CD161+ NK cells were gated from total NK cells. *Abbreviations:* CM, central memory; EM, effector memory; TEMRA, effector memory re-expressing CD45RA; SM, switched memory; NSM, non-switched memory; DN, double negative; Mo-MDSCs, Monocytic myeloid derived suppressor cells

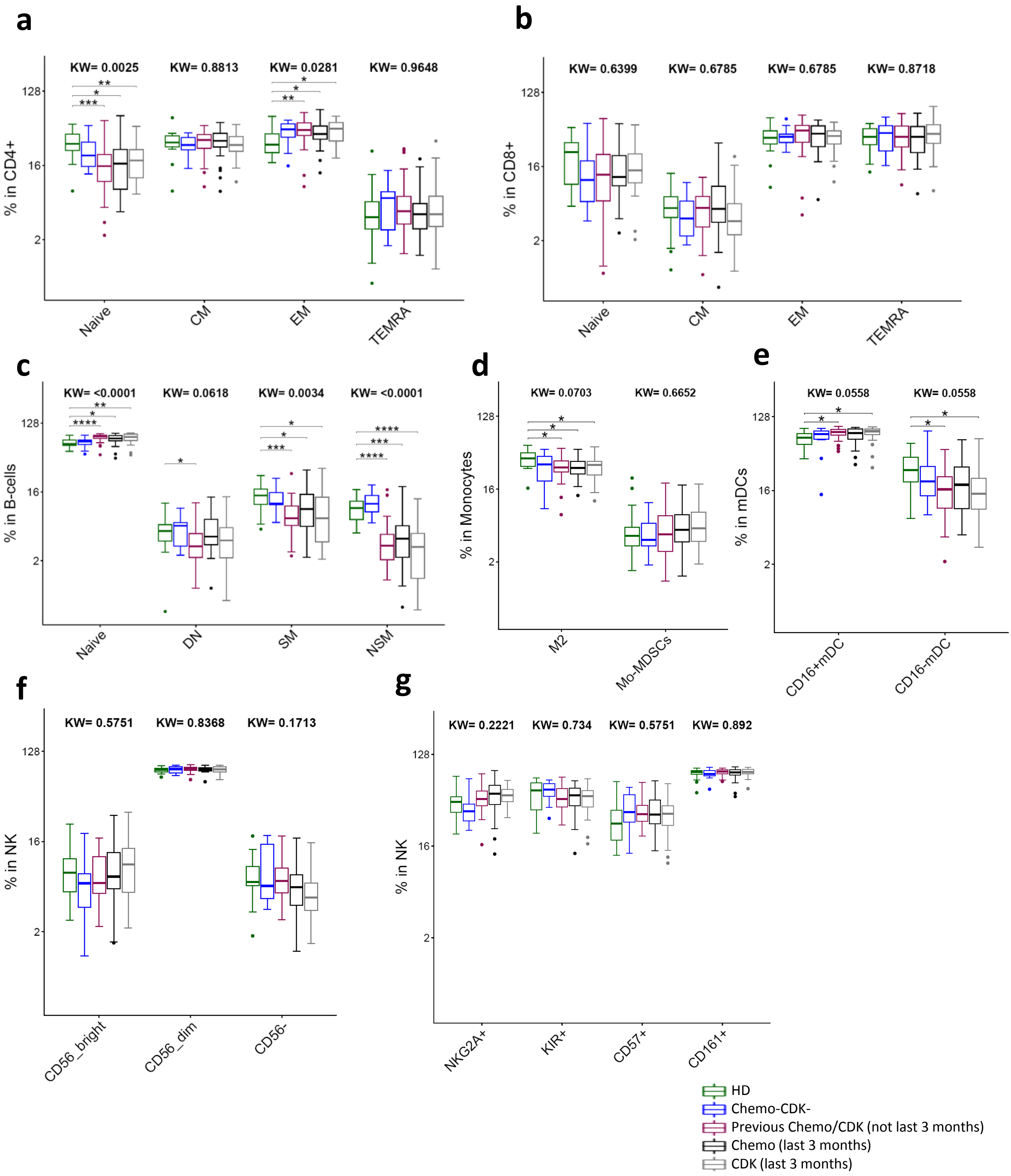

Supplementary Figure 3. Effect of previous chemotherapy and CDK inhibitors on differentiation subsets of immune cells in BC patients. **(a)** CD4+T **(b)** CD8+T **(c)** B-cells **(d)** Monocytes **(e)** mDCs **(f-g)** NK cells. Received no chemotherapy/CDK inhibitors (Chemo-CDK-; n=10), received chemotherapy/CDK inhibitors >3 months before sample collection [Previous Chemo/CDK (not last 3 months); n=40], received chemotherapy within 3 months before sample collection [Chemo (last 3 months); n=26], received CDK inhibitors within 3 months before sample collection [CDK (last 3 months); n=28]. Groups were compared by kruskal wallis test, followed by pair-wise comparisons using Wilcoxon Mann-Whitney Rank Sum Test with HD as a reference group. FDR-adjusted p-value (p.adj) < 0.05 was considered statistically significant. . *p.adj<0.05, **p.adj<0.01, ***p.adj<0.001, ****p.adj<0.0001.

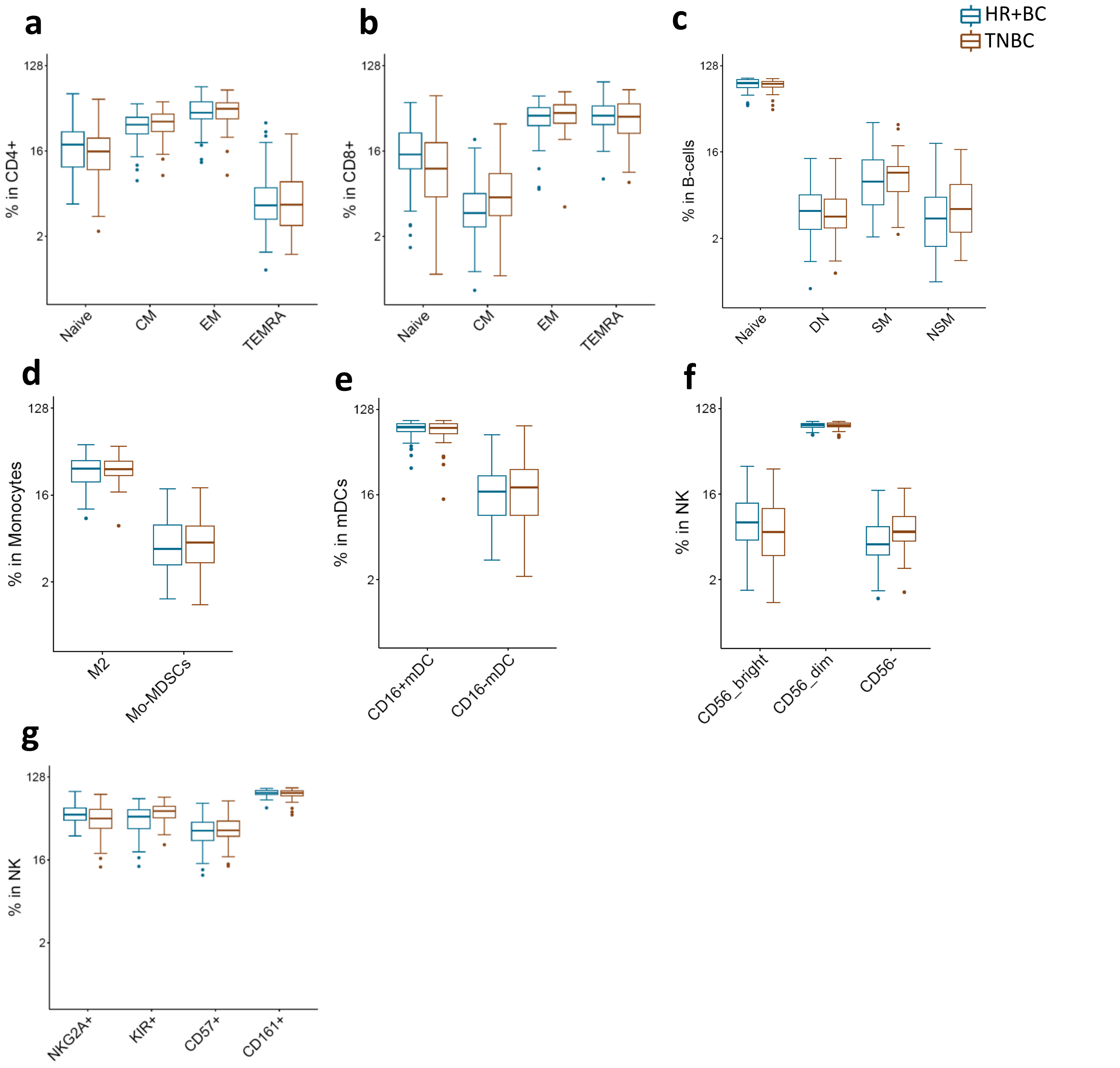

Supplementary Figure 4. Abundance of differentiation subsets of immune cells between HR+BC and TNBC patients. **(a)** CD4+T **(b)** CD8+T **(c)** B-cells (**d)** Monocytes **(e)** mDCs **(f-g)** NK cells. Groups were compared by Wilcoxon Mann-Whitney Rank Sum Test. FDR-adjusted p-value (p.adj) < 0.05 was considered statistically significant. . *p.adj<0.05. HR+BC: n=57. TNBC: n=47.

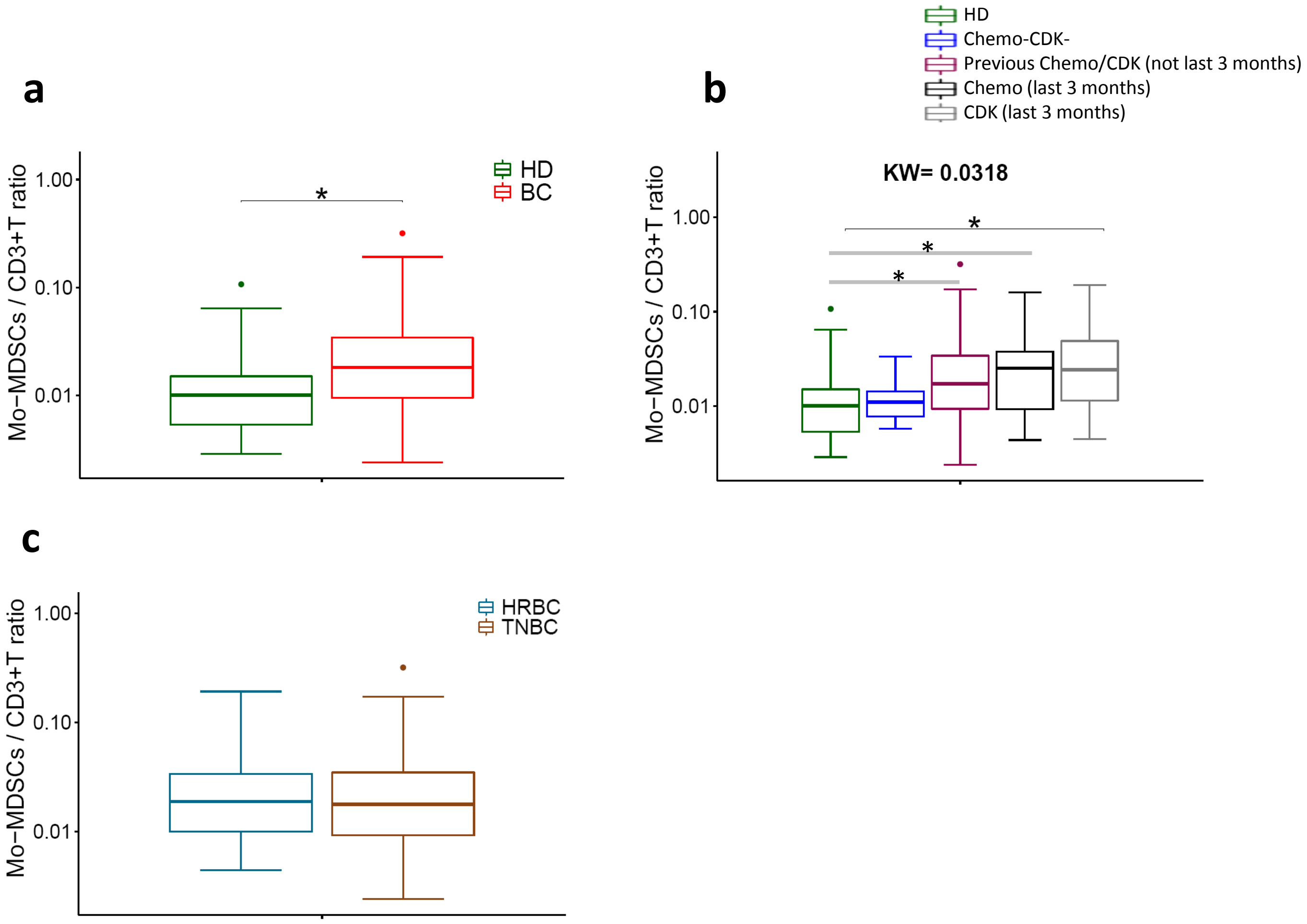

Supplementary Figure 5. Comparison of Mo-MDSCs/CD3+T cells ratio. **(a)** Mo-MDSCs to CD3+T cells ratio between HD (n=20) and BC patients (n=104). Wilcoxon Mann-Whitney Rank Sum Test; *p<0.05 **(b)** Effect of previous chemotherapy and CDK inhibitors on Mo-MDSCs to CD3+T cells ratio**.** Received no chemotherapy/CDK inhibitors (Chemo-CDK-; n=10), received chemotherapy/CDK inhibitors >3 months before sample collection [Previous Chemo/CDK (not last 3 months); n=40], received chemotherapy within 3 months before sample collection [Chemo (last 3 months); n=26], received CDK inhibitors within 3 months before sample collection [CDK (last 3 months); n=28]. **(c)** Mo-MDSCs to CD3+T cells ratio between HRBC (n=57) and TNBC patients (n=47). Kruskal wallis test, followed by pair-wise comparisons using Wilcoxon Mann-Whitney Rank Sum Test with HD as a reference group. FDR-adjusted p-value (p.adj) < 0.05 was considered statistically significant. *p.adj<0.05, **p.adj<0.01, ***p.adj<0.001, ****p.adj<0.0001.

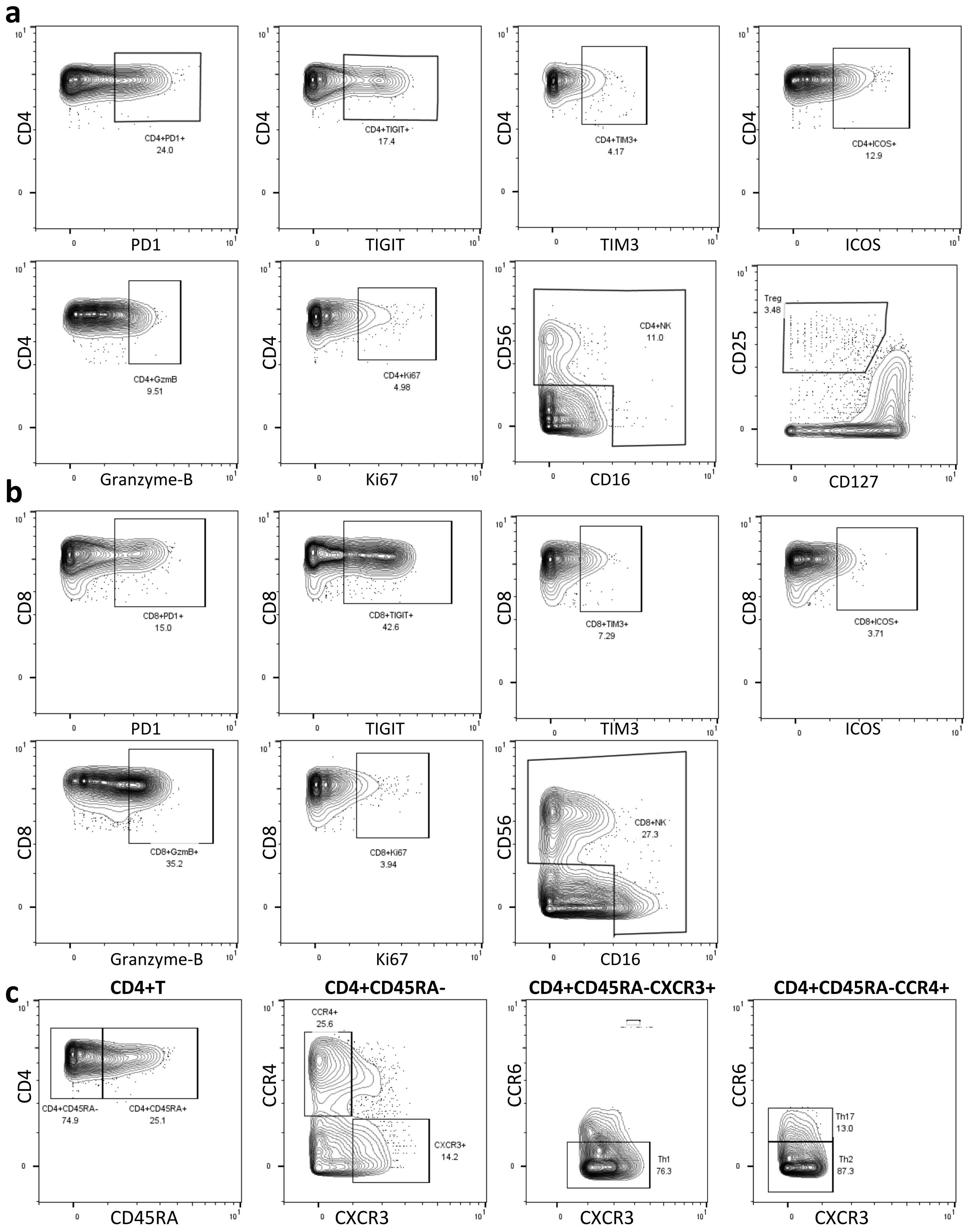

Supplementary Figure 6. Gating scheme for phenotypic and functional markers corresponding to figure 3 and regulatory T cells (Tregs) by mass cytometry. **(a)** Representative staining of PD1, TIGIT, TIM3, ICOS, Granzyme-B, Ki67, NKT and Treg markers on CD4+T cells. **(b)** Representative staining of PD1, TIGIT, TIM3, ICOS, Granzyme-B, Ki67, and NKT markers on CD8+T cells **(c)** Identification of Th subsets in CD4+T cells. *Abbreviations:* NKT, natural killer T cells

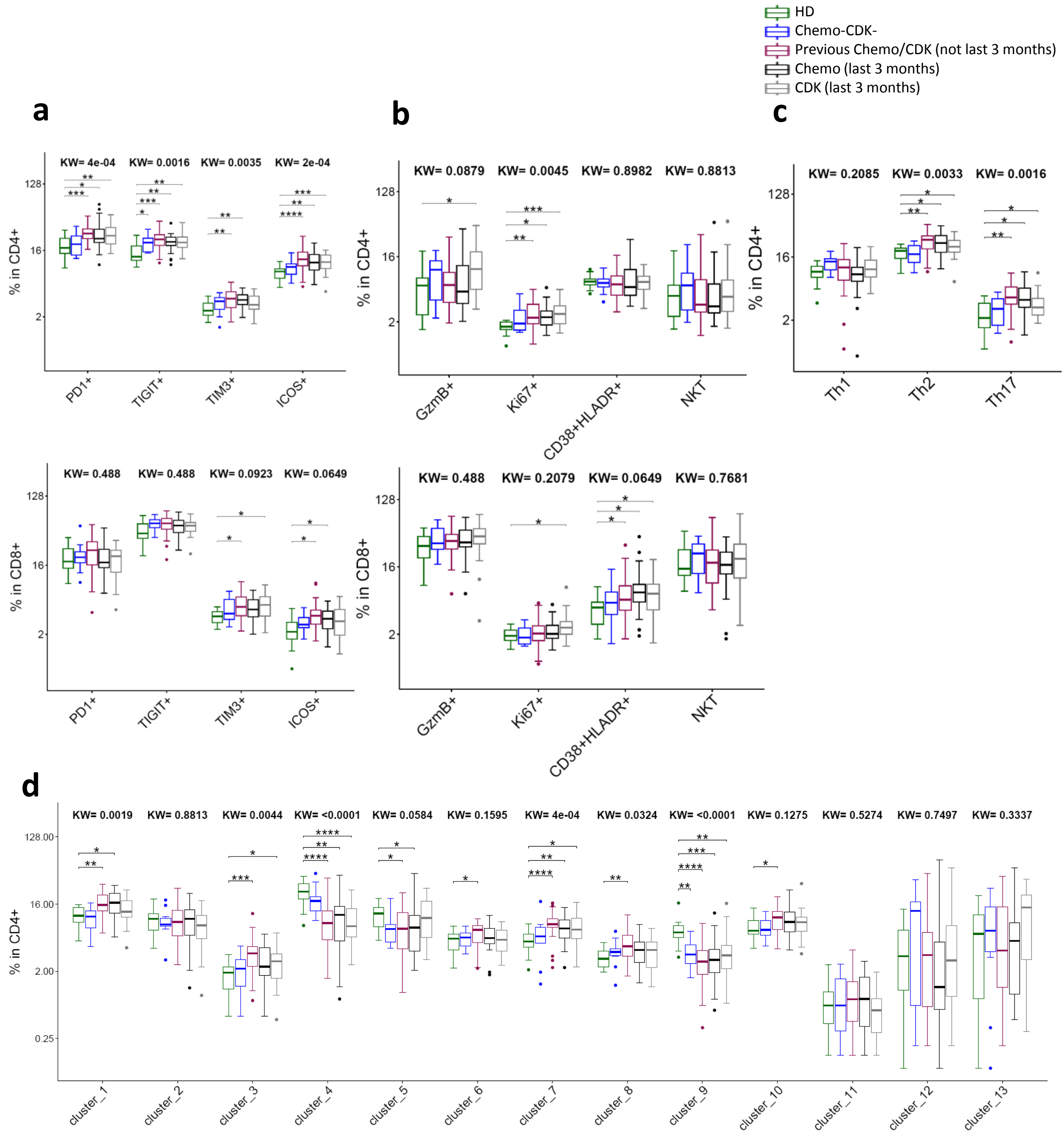

Supplementary Figure 7. Effect of previous chemotherapy and CDK inhibitors on phenotype of T cells in BC patients. Expression of immune checkpoint receptors **(a)** and functional/phenotypic markers (**b**) in CD4+ and CD8+ T cells. **(c)** Abundance of Th1/Th2/Th17 subsets in CD4+ T cells. **(d)** Abundance of CD4+T cell clusters identified by phenograph. Received no chemotherapy/CDK inhibitors (Chemo-CDK-; n=10), received chemotherapy/CDK inhibitors >3 months before sample collection [Previous Chemo/CDK (not last 3 months); n=40], received chemotherapy within 3 months before sample collection [Chemo (last 3 months); n=26], received CDK inhibitors within 3 months before sample collection [CDK (last 3 months); n=28]. Groups were compared by kruskal wallis test, followed by pair-wise comparisons using Wilcoxon Mann-Whitney Rank Sum Test with HD as a reference group. FDR-adjusted p-value (p.adj) < 0.05 was considered statistically significant. *p.adj<0.05, **p.adj<0.01, ***p.adj<0.001, ****p.adj<0.0001.

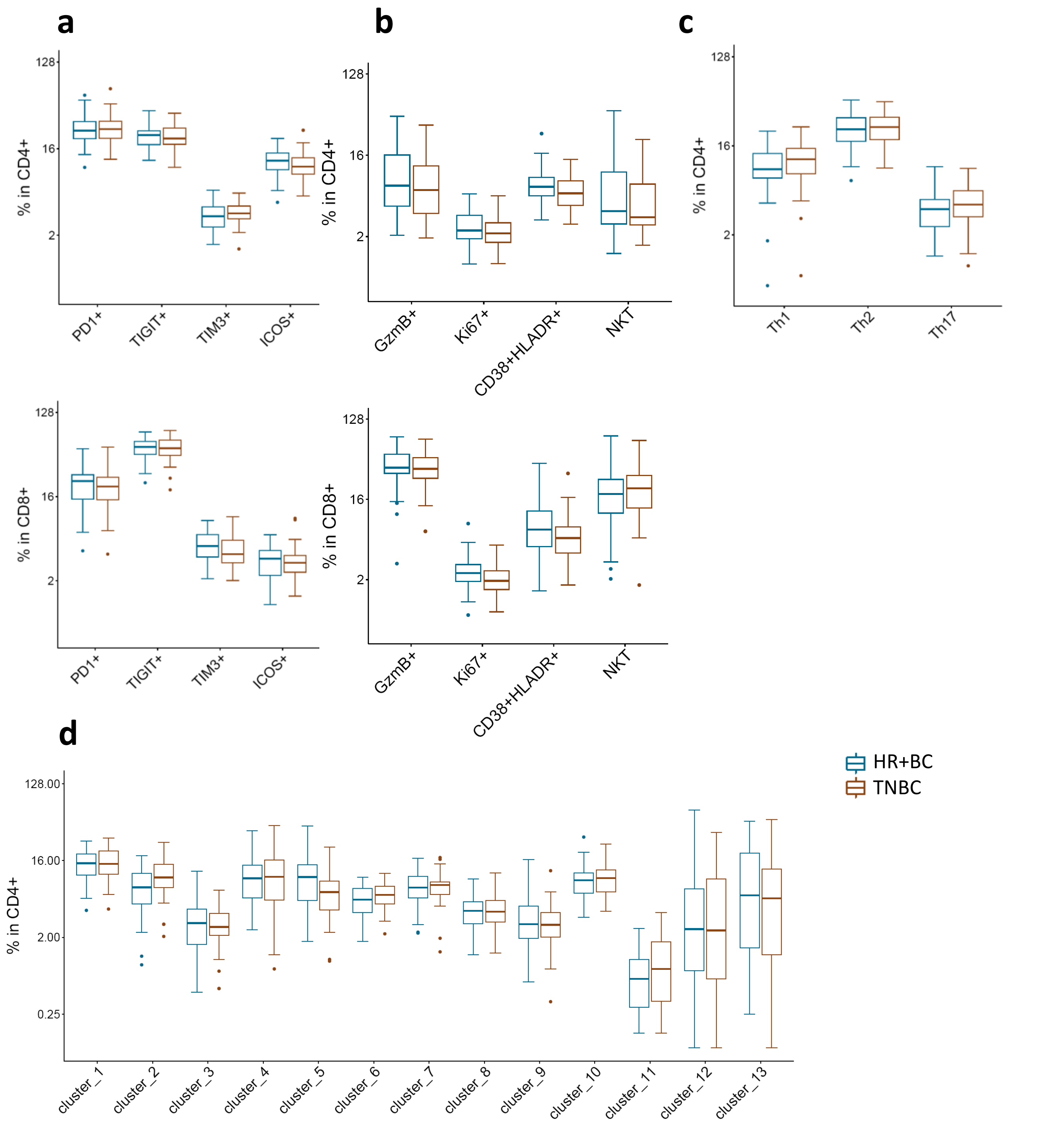

Supplementary Figure 8. Comparison of T cell phenotype between HR+BC and TNBC patients. Expression of immune checkpoint receptors **(a)** and functional/phenotypic markers (**b**) in CD4+ and CD8+ T cells. **(c)** Abundance of Th1/Th2/Th17 subsets in CD4+ T cells. **(d)** Comparative abundance of different CD4+ immune cell clusters as identified by phenograph on UMAP between HR+BC and TNBC patients. Groups were compared by Wilcoxon Mann-Whitney Rank Sum Test. FDR-adjusted p-value (p.adj) < 0.05 was considered statistically significant. *p.adj<0.05, **p.adj<0.01. HR+BC: n=57. TNBC: n=47.

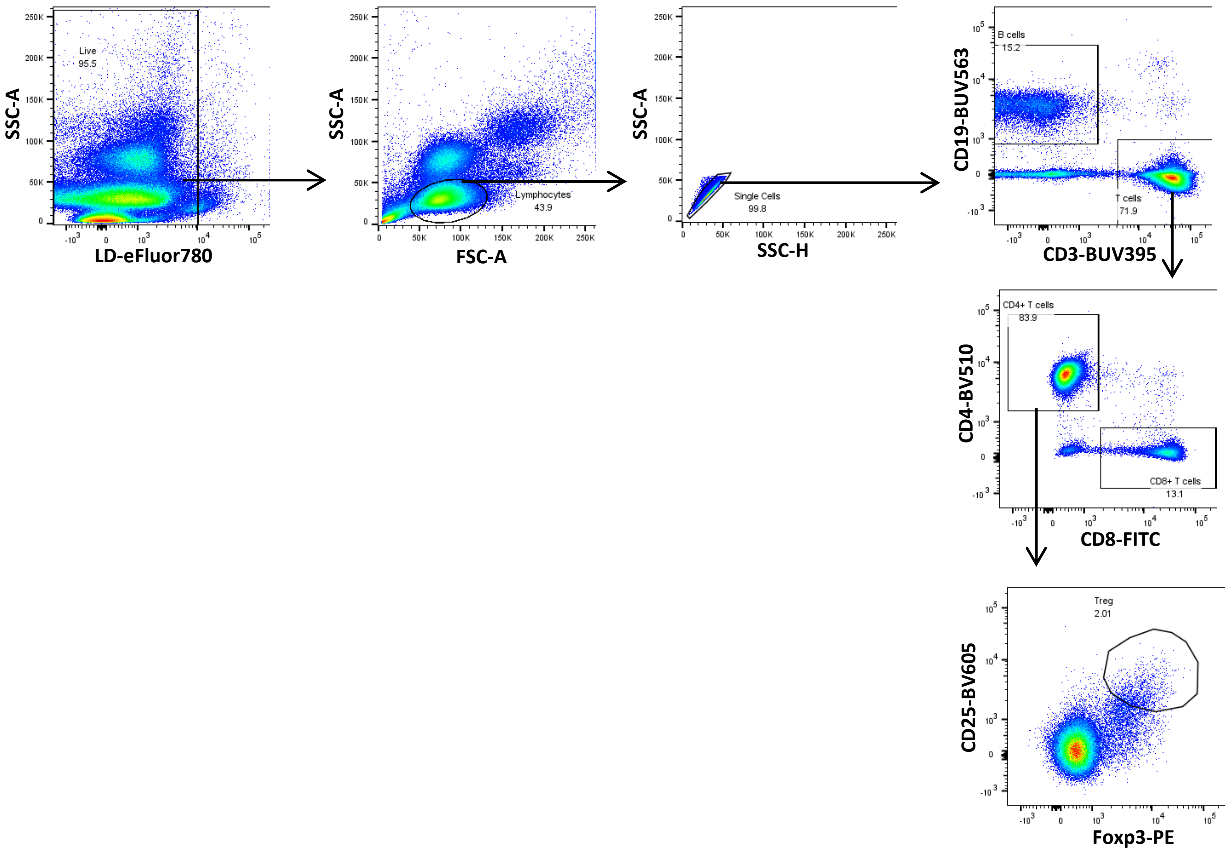

Supplementary Figure 9. Flow cytometry gating strategy for identifying regulatory T cells (Tregs) in PBMCs. Pseudocolor dot plots of PBMCs from a representative patient showing the gating strategy used to identify the Tregs by flow cytometry. Total PBMCs were gated using L/D (fixable viabilty dye eFluor780) and lymphocytes were identified by FSC-A and SSC-A and cell doublets were excluded based on SSCA and SSC-H. Tregs were defined as $CD3^{+}CD4^{+}$ $CD25^{+}Foxp3^{+}$.

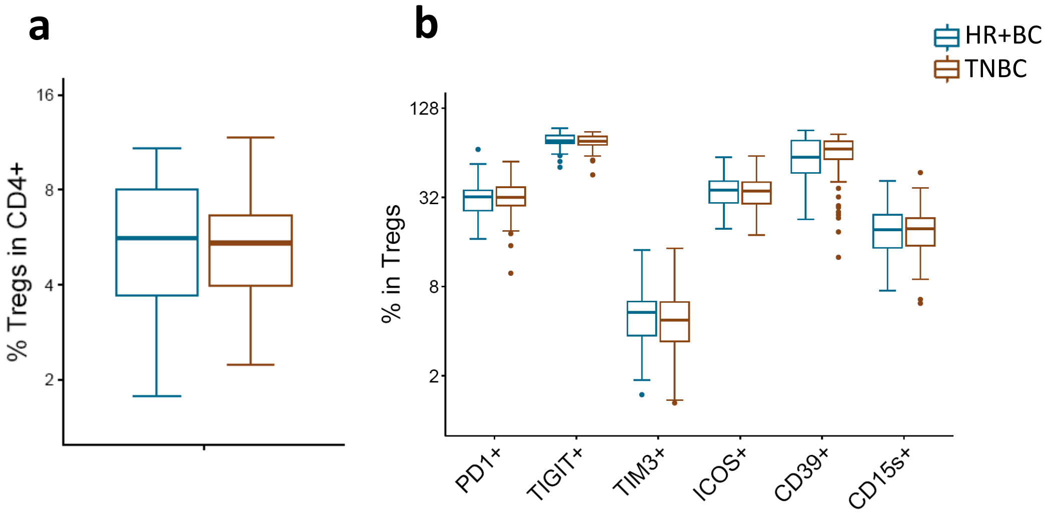

Supplementary Figure 10. Comparison of Treg phenotype between HR+BC and TNBC patients. **(a)** Abundance of Tregs (CD25+CD127-) in CD4 compartment in HR+BC and TNBC patients. **(b)** Expression of various immune checkpoints and functional markers on Tregs in HR+BC and TNBC patients. Two groups were compared by Wilcoxon Mann-Whitney Rank Sum Test. FDR-adjusted p-value (p.adj) < 0.05 was considered statistically significant. *p.adj<0.05, **p.adj<0.01, ***p.adj<0.001, ****p.adj<0.0001. HR+BC: n=57. TNBC: n=47.

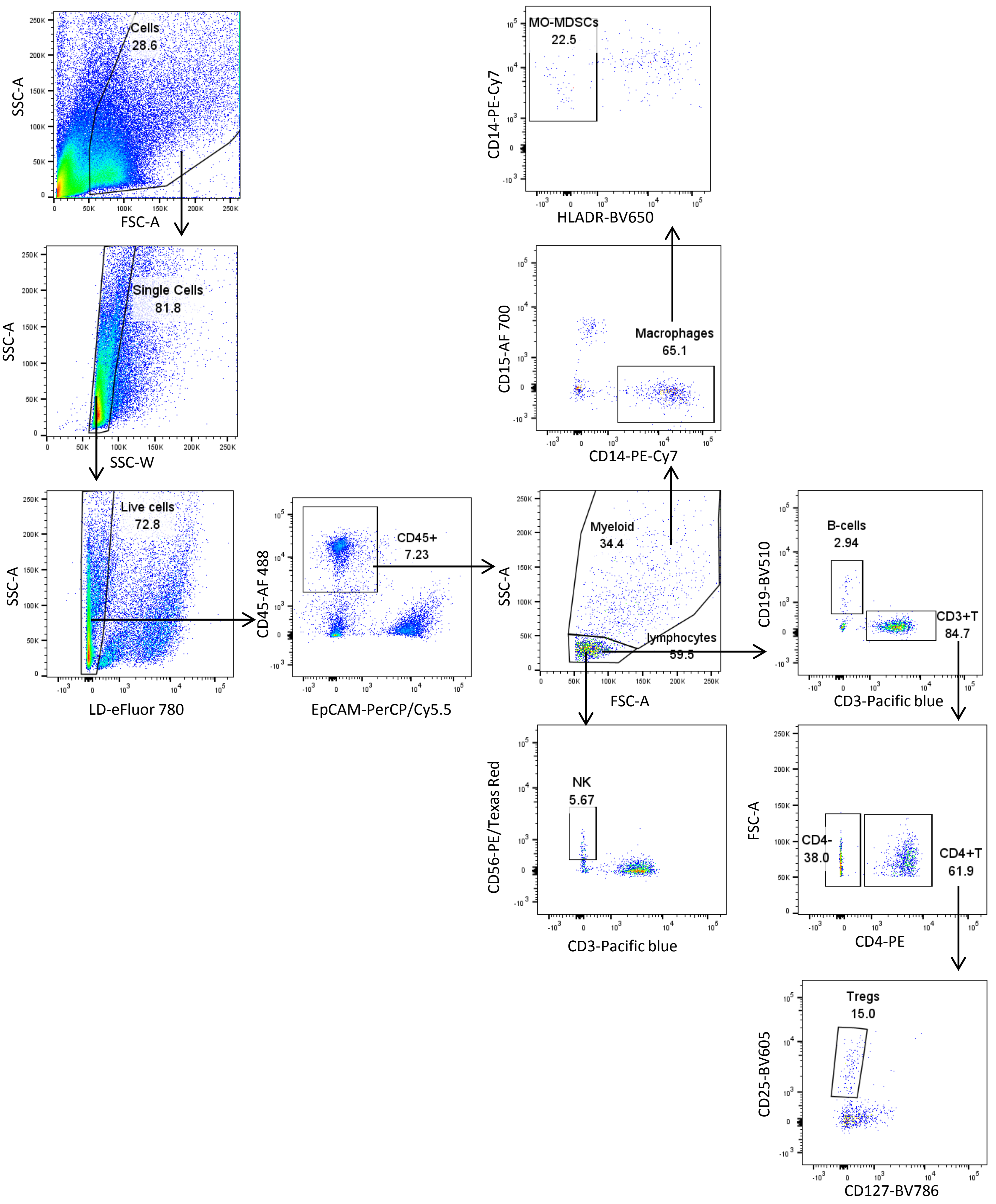

Supplementary Figure 11. Flow cytometry gating scheme for identifying immune cell subsets in tumor biopsies of metastatic breast cancer patients. Immune cell subsets are defined as follows: CD4+ T cells (CD3+CD4+CD8-), CD3+CD4- T cells (CD3+CD4-), Regulatory T cells (CD3+CD4+CD25+CD127-), B-cells (CD3-CD19+), NK cells (CD3-CD56+), Macrophages (CD14+CD15-) and Mo-MDSCs (CD14+HLADR-).
